# Investigating the accuracy of tropical woody stem CO_2_ efflux estimates: scaling methods, and vertical and diel variation

**DOI:** 10.1111/nph.70122

**Published:** 2025-04-03

**Authors:** Maria B. Mills, Alexander Shenkin, Phil Wilkes, Mathias Disney, Susan Page, Juan Carlos Berrio, Jörg Kaduk, Yadvinder Malhi, Rolando Robert, Reuben Nilus, Terhi Riutta

**Affiliations:** ^1^ School of Geography Geology and the Environment University of Leicester Leicester LE2 1TF UK; ^2^ School of Informatics, Computing, and Cyber Systems Northern Arizona University Flagstaff AZ 86011 USA; ^3^ Environmental Change Institute, School of Geography and the Environment University of Oxford Oxford OX1 3QY UK; ^4^ Kew Wakehurst Ardingly West Sussex RH17 6TN UK; ^5^ Department of Geography University College London Gower Street London WC1E 6BT UK; ^6^ NERC National Centre for Earth Observation (NCEO) UCL Gower Street London WC1E 6BT UK; ^7^ Sabah Forestry Department Forest Research Centre Sandakan Sabah 90715 Malaysia; ^8^ Department of Life Sciences Imperial College London London SL5 7PY UK; ^9^ UK Centre for Ecology and Hydrology Wallingford OX10 8BB UK

**Keywords:** carbon fluxes, diel respiration, respiratory scaling, stem respiration, surface area allometries, terrestrial LiDAR, tropical forest

## Abstract

- Stem CO_2_ efflux (EA) significantly contributes to autotrophic and ecosystem respiration in tropical forests, but field methodologies often introduce biases and uncertainty. This study evaluates these biases and their impact on scaling EA at the stand‐level.
- Diel and vertical patterns of EA were investigated, along with the accuracy of estimating stem surface area from allometric equations vs terrestrial light dection and ranging (LiDAR) scanning (TLS) in Maliau Basin Conservation Area, Sabah, Malaysian Borneo.
- Diel EA exhibited no uniform pattern due to inter‐tree variability, but results suggest measuring EA before 15:00 h. EA was significantly higher on buttresses and above the first major branching point, but vertical variations in EA did not impact stand‐level EA when stem surface area was accurately estimated. Allometric equations underestimated total stem surface area by *c.* 40% compared with TLS, but applying a site‐specific correction factor yielded a similar stand‐level EA and total stem surface area to TLS.
- This study provides guidance for measuring EA in the field and suggests that measuring at one time point and one height along the stem can produce accurate results if conducted using the correct time frame and if stem surface area is accurately estimated.

## Introduction

Stem respiration is CO_2_ produced by respiration inside the stem (Bowman *et al*., 2008) due to the metabolic activity of woody cells during plant growth and maintenance (Malhi *et al*., 2009). As tree stems consist of several layers of metabolically active tissue, it can be difficult to measure the respiration rate of individual tissues below the stem surface (Teskey *et al*., 2008; Trumbore *et al*., 2013) or be certain of the origin of diffused CO_2_ (Salomón *et al*., 2024). CO_2_ may be transported away from the origin site upwards in the xylem (Hölttä & Kolari, 2009), and CO_2_ originating elsewhere, such as the root system, can also be transported upwards and diffused out within the stem and upper canopy (Teskey *et al*., 2008; Trumbore *et al*., 2013). Woody stem CO_2_ efflux to the atmosphere (EA) is the CO_2_ efflux measured at the stem surface and is widely used as a proxy for stem respiration. EA is methodologically simpler to quantify and is largely a measure of the *in situ* autotrophic respiration of the biologically active outer layer of the stem (Robertson *et al*., 2010), although it is acknowledged that EA does not necessarily equal stem respiration.

In tropical rainforests, EA is a substantial contributor to the carbon budget, accounting for 23–42% of autotrophic respiration (Mills *et al*., 2023) and *c*. 13–25% of total ecosystem respiration (Chambers *et al*., 2004; Cavaleri *et al*., 2006; Malhi *et al*., 2009; Mills *et al*., 2023). EA is highly reflective of tree metabolism and can provide vital information about forest allocation and investment strategies (Mills *et al*., 2024), and response to environmental pressures such as drought (Rowland *et al*., 2018) and elevational gradients (Zach *et al*., 2008, 2010; Robertson *et al*., 2010). Yet, current methods of estimating EA allow for a large potential for bias and uncertainty. Field studies assume that EA is constant with height and diurnally, and observations at the tree‐level are upscaled to the 1‐ha forest plot using estimates of woody stem surface area from allometric equations. In tropical forests particularly, the assumption of constant EA is largely for methodological simplicity.

Current methods for estimating EA sample are to take a proportion of trees of varying sizes and species per plot (usually 40–50 trees per 1‐ha plot) and to take one measurement per tree at a convenient measurement height, typically between 1.1 and 1.5 m from the ground (termed breast height), usually during office hours (09:00 h–15:00 h). Although logistically simpler, especially in remote tropical forests, measuring at one timestamp assumes that EA does not vary over 24 h, which could result in biases according to the time measurements were conducted or be reflective of other forms of CO_2_ consumption and transport (Teskey *et al*., 2008; Angert *et al*., 2012). Temperature is a dominant driver of stem respiration (Stockfors, 2000; Zha *et al*., 2004; Saveyn *et al*., 2008) and has previously been used to model stem respiration as a function of the Q_10_ parameter (Hölttä & Kolari, 2009; Darenova *et al*., 2019). These thermal‐driven variations in EA have largely been reported outside the tropics, with contradictory findings within the tropics where thermal variations are limited in comparison with other areas. In tropical regions, diel variation has been observed previously in Amazonia (Kunert, 2018; Jardine *et al*., 2022), Thailand (Marler & Lindström, 2020), Northern China (Yang *et al*., 2014), and Beijing (Han *et al*., 2017). Other studies have, however, reported no diel pattern in south Ecuador (Zach *et al*., 2008) and limited variation in eastern Amazonia (Rowland *et al*., 2018). Such studies have been limited in their sample size (Yang *et al*., 2014; Kunert, 2018; Jardine *et al*., 2022) due to logistical constraints with equipment and accessibility (Kunert, 2018), which is particularly challenging in remote locations. There is also a trade‐off in EA studies between temporal and spatial resolution, for example sampling a larger number of trees and plots vs the frequency of visits and total duration of the study (Mills *et al*., 2024). Given such constraints in physically measuring diel EA, the question remains: What is the most representative time of day to measure EA? (Kunert, 2018).

Similarly, there are methodological constraints on measuring vertical EA, especially in tropical regions where trees are typically tall in stature and forests are dense. Due to this, EA is typically assumed constant and measured at breast height only. Research on vertical EA in tropical forests has produced conflicting results. Studies have reported that EA varies with stem height (Cavaleri *et al*., 2006; Kunert, 2018), whilst other studies found no clear pattern of vertical variation (Katayama *et al*., 2014, 2019), or a decrease with stem height (Asao *et al*., 2015). Studies conducted outside the tropics have generally reported an increase in EA with height (Damesin *et al*., 2002; Araki *et al*., 2010; Tarvainen *et al*., 2014; Han *et al*., 2017; Martínez‐García *et al*., 2017). It is expected that EA would vary vertically along the stem due to gradients in stem temperature (Kunert, 2018), stem growth (Araki *et al*., 2010, 2015; Tarvainen *et al*., 2014), and variations in bark depth (Katayama *et al*., 2014). Variations with height may also be a result of an increase in diffused CO_2_ being transported upwards and diffusing out through the thinner bark in the canopy (Bowman *et al*., 2005). This may be further influenced by differences in solar radiation in the canopy affecting bark water content and subsequent CO_2_ diffusion (Hölttä & Kolari, 2009), resulting in higher variation of EA in the canopy compared with at 1.1‐m height (Katayama *et al*., 2014). Due to the difficulties in measuring within the canopy, studies measuring vertical variation are often limited in sample size (Cavaleri *et al*., 2006; Katayama *et al*., 2014; Asao *et al*., 2015; Kunert, 2018), and physical sampling can involve assembling towers and scaffolding (Katayama *et al*., 2014; Kunert, 2018). In combination, these limitations make it challenging to quantify variation across a wider range of tree sizes and species (Katayama *et al*., 2019). Given the logistical difficulty of measuring EA with height, it is important to quantify to what extent traditional point sample measurements at breast height are representative of whole tree‐level EA. Previous work in Borneo has examined this and found that tree‐level estimates of EA using measurements at breast height vs with vertical variation were similar (Katayama *et al*., 2014, 2019). This, however, does not account for any bias or uncertainties involved in how EA is upscaled to tree and stand levels.

In order to provide a stand‐level estimate of EA, the average EA of the sampled trees is typically upscaled to the 1‐ha plot to give a stand‐level estimate of EA, usually annually. Sapwood area (sapwood vertical plane) and stem surface area have previously been used as scaling parameters; scaling to surface area is deemed most accurate and convenient for scaling at the ecosystem level (Chambers *et al*., 2004; Robertson *et al*., 2010). In tropical forests specifically, sapwood area is poorly defined and there are interspecific variations in these species' diverse ecosystems (Katayama *et al*., 2019). Estimates of stem surface area are computed from allometric equations based on tree diameter, which are typically derived from a small number of destructively sampled trees – particularly within South America (Chave *et al*., 2005, 2014). This can be problematic due to the biogeographic differences in stem diameter–height relationships that vary by both forest type and region (Feldpausch *et al*., 2011; Meir *et al*., 2017), complex tree shapes, for example buttresses (Meir *et al*., 2017), and variations in wood density (Patiño *et al*., 2009). In Malaysia particularly, stand basal area was found to be an important driver of variations in stem height–diameter allometries, and forests with greater basal area tended to have taller trees at any given diameter (Feldpausch *et al*., 2011). Research has acknowledged the inherent bias in surface area equations and assigns a 10% error for the purposes of error propagation and uncertainty (Robertson *et al*., 2010). Recent developments in terrestrial light detection and ranging (LiDAR) have provided the opportunity to address these complexities by enabling the quantification of tree form (Meir *et al*., 2017). By comparing woody stem surface area estimates derived from terrestrial LiDAR with traditional estimates derived from allometric equations, it is possible to estimate the accuracy of current estimates of stand‐level stem surface area, and the consequences for respiratory scaling.

Collectively, several sources of bias contribute to the uncertainty in EA measurements; it is, therefore, important to quantify such biases and investigate forms of mitigation. This study, therefore, investigates diel and vertical patterns of EA and stem surface area scaling accuracy using the Maliau Basin Conservation Area (MBCA) in Sabah, Malaysian Borneo, as a case study. Specifically, this study aimed to:

1. determine whether EA has a diel cycle and suggest a representative time frame to conduct measurements;
2. quantify whether EA varies vertically and compare estimates accounting for vertical variations to the estimates from breast height only;
3. compare stem surface area estimates from allometric equations that use tree diameter with estimates derived from terrestrial LiDAR for scaling EA from tree‐level to stand‐level; and
4. determine the consequence of these biases for the scaling of EA to stand‐level and suggest forms of mitigation.

## Materials and Methods

### Study site

This study was conducted within the MBCA in Sabah, Malaysian Borneo, within MLA‐01 Maliau Belian plot, which is a 1‐ha intensive Global Ecosystem Monitoring plot (Marthews *et al*., 2014; Malhi *et al*., 2021). This area is an old‐growth, lowland, dipterocarp‐dominated, humid tropical forest with no evidence of human disturbance or logging. The climate is moist tropical, with no regular seasonal periods (Katayama *et al*., 2016). Average daily temperature per month in MBCA in 2023 ranged from 24.6 to 26.9°C, and average daily precipitation per month ranged from 5.28 to 26.23 (Yayasan Sabah Group, unpublished data). The 1‐ha study plot, which is divided into 25 subplots of 20 m × 20 m, has been subject to biannual forest census and intensive carbon monitoring (Riutta *et al*., 2018, 2021; Mills *et al*., 2023). Stand‐level EA of this plot has been estimated at 7.82 ± 0.34 Mg C ha^−1^ yr^−1^ based on monthly data from 2011 to 2019, of which 7.13 ± 4.38 Mg C ha^−1^ yr^−1^ is allocated to maintenance respiration and 0.70 ± 4.38 Mg C ha^−1^ yr^−1^ to growth respiration (Mills *et al*., 2024). The three most abundant tree genera are *Dryobalanops, Rubroshorea*, and *Eusideroxylon* of 144 identified species (Fig. S1). Within the plot, the basal area is 41.6 ± 3.59 m^2^/ha and there are 397 trees (> 10 cm diameter; Fig. S2), of which 47 had a diameter at breast height (DBH) of > 50 cm (Riutta *et al*., 2018).

### Diel EA measurements

The field campaign (for both vertical and diel EA measurements) took place during May 2023. Across the measurement period, conditions were generally dry during the daytime, with showers throughout the evenings (Fig. S3). Diel EA was measured within four subplots on five trees per subplot over 9 consecutive days. Measurements were taken continuously over 48 h per subplot, except for subplot 23 (group A), which was measured for 72 h. Subplots were selected based on their accessibility and variation of stem sizes. After the five trees within each selected subplot had been measured for 48 h, the chambers were removed, and the flux equipment (Fig. S4) was manually relocated to the next subplot. The flux equipment and chambers were then connected to the next set of five trees, and a new measurement cycle commenced. The DBH (1.3 m) of the trees selected ranged from 13 to 208 cm and height from 11 to 45 m (Fig. S2). Trees sampled were of 16 different species of seven different genera (Fig. S1), with the most common family being Dipterocarpaceae (50% of sampled trees); trees of the Dipterocarpaceae made up *c*. 62% of basal area within the 1‐ha plot. Temperature and humidity were measured in each subplot at the stem surface continuously over the measurement period using Tinytag data loggers (TGP‐4500; Gemini). Over the study period, temperature ranged from 22.4 to 30.9°C, with an average temperature of 24.12 ± 0.13°C, and relative humidity ranged from 68.6 to 100%, with an average of 88.41 ± 1.44% (Fig. S3). There was no significant difference in temperature (ANOVA; *P* = 0.07) or relative humidity (ANOVA; *P* = 0.35) between measurement days.

On each sampled stem, a platic polyvinyl chloride (PVC) collar 7 cm in length with a 10.6 cm internal diameter was installed at a height of 1.1 m with silicone sealant (Fig. S4). Before installation, any mosses, epiphytes, or insect nests were removed. EA was measured every hour using a LiCOR Li8100A infrared gas analyser (IRGA) and LiCOR Li8150 multiplexer with 15 m extension cables, powered by a 100‐ah car battery. The equipment was configured to operate as a closed, self‐flushing multiplexed system (LiCOR, 2019). To create a closed system, plastic caps with an 11 cm diameter, fitted with in‐and‐out push fittings, were secured to the plastic collars and connected to the LiCOR system using 15‐m extension cables (Fig. S4). Each measurement duration was 3 min, with a 90‐s dead band and flushed with ambient air between observations. Over the 3‐min interval, CO_2_ accumulates inside the system and the CO_2_ flux is calculated as the linear change in CO_2_ concentration (LiCOR, 2015):

$$\mathrm{EA}=\frac{10V\;P_{0}\left(1-\frac{W_{0}}{1000}\right)}{\mathrm{RS}\left(T_{0}+273.15\right)\;}\;\frac{{\partial C}^{\prime }}{\partial t}$$

where EA is the CO_2_ efflux rate (μmol m^−2^ s^−1^), V is the volume (cm^3^; chambers and extension cables), *P*_0_ is the initial pressure (kPa), *W*_0_ is the initial water vapour mole fraction (mmol mol^−1^), *R* is the gas constant (8.314 J/(mol·K)), *S* is the woody stem surface area (cm^2^), *T*_0_ is the initial air temperature (°C), and $\frac{{\partial C}^{\prime }}{\partial t}$ is the initial rate of change in water‐corrected CO_2_ mole fraction (μmol mol^−1^). EA was then scaled to give EA in mg C m^−2^ h^–1^. Data were subject to quality control and outlier detection and removal of data in which there had been incidents of leakage, mechanical error, or outside logical bounds. Outlier detection and removal was conducted per tree. Data that were 1.5 times outside the interquartile range (below the first quartile or above the third quartile) were removed (*n* = 13 observations). Due to battery constraints, not all cycles were complete or continuous for every tree. The longest continuous cycle was *c*. 22 h and the shortest *c*. 11 h. After quality control, 18 trees were sampled and observations per tree ranged from 14 to 46, and per hour ranged from 7 to 35 observations of EA. The final diel dataset consisted of 579 individual observations of diel EA.

### Vertical EA measurements

Vertical EA was measured on 13 trees by a team of tree climbers, with a minimum of four height intervals (range 4–7) per tree that were *c*. 5 m or *c*. 10 m apart, dependent on tree height (Fig. S5). Trees ranged from 42.6 to 105.5 cm DBH (Fig. S2) and from 27 to 65 m in height, with the highest EA measurement at 50 m. Eleven different species of six different families were sampled (Fig. S1), with the most dominant family being Dipterocarpaceae (47% of sampled trees). All measurements were taken on the main stem, or on the first major branch if the tree displayed a Y branching architecture. The final dataset consisted of 68 observations of vertical EA, with six measurements on buttresses, 49 on the stem, and 13 on the stem above the first branching point. The smallest diameter at point of measurement was 27.0 cm and the largest 105.8 cm.

Safety and accessibility determined which trees could be sampled, and trees were selected which were also sampled in the 2018 terrestrial LiDAR campaign. Sampling took 35 min to 1 h 25 min per tree, and all measurements were taken between 09:00 h and 15:30 h across five consecutive days. At each sampling point, a PVC collar 10 cm in length with a 10.6 cm internal diameter was attached to the tree using ratchet straps and hose clips, and modelling clay was used to create an airtight seal around the collar (Fig. S5). Before commencing each measurement, the chamber was flushed and collar fanned to remove stagnant air, and the collar was checked for leakage. To measure EA, an infrared gas analyser (EGM‐4; PP Systems) and soil respiration chamber (SCR‐1; PP Systems) were employed, and a custom ring adapter of 11 cm diameter and 3.5 cm height was fitted to the chamber to match the diameter of the collars and enable an airtight seal to avoid leakage (Marthews *et al*., 2014). The chamber was placed onto the collar, and CO_2_ efflux was measured for 120 s. Over the 120 s, CO_2_ accumulates in the chamber, and the uncorrected CO_2_ flux (*R*_u_; ppm s^−1^) is calculated by the IRGA by fitting a linear regression between CO_2_ concentration and time (mean *R* ^2^ = 0.954). The CO_2_ flux is then calculated using the ideal gas law (Marthews *et al*., 2014). At each sampling point, measurement height on the tree (using a Nikon Forestry Pro) and stem surface temperature (using a Kestrel 4500) were recorded, and diameter was recorded at the first and last measurement. Data were corrected to 25°C assuming a Q_10_ of 2.0 (Cavaleri *et al*., 2006). Average air temperature across all sampling points was 29.24 ± 0.22°C and ranged from 24.9 to 31.8°C, and average air temperature range across a tree's vertical profile was 2.12 ± 0.436°C (range 0.3–4.9°C).

### Terrestrial LiDAR

Terrestrial LiDAR data were acquired in 2018 with a RIEGL VZ‐400 scanner (RIEGL Laser Measurement Systems GmbH, Horn, Austria) whereby a set of scans were captured on a regular grid (Wilkes *et al*., 2017). Scan patterns were conducted in 10‐m grid patterns, and two scans were acquired at each scan position whereby the scanner rotation axis was orientated perpendicular then parallel to the ground. To enable coregistration, manually placed reflectors were used as tie point between scan positions (Wilkes *et al*., 2017). Postprocessing and coregistration were conducted within the riscan pro software (v.2.9). Manual segmentation of trees was performed within CloudCompare (https://www.danielgm.net/cc). Point clouds were subject to semantic segmentation into leaf and wood points using TLSeparation python package (Vicari *et al*., 2019). Quantitative structure models (QSM) for each tree were then created using wood classified points only using treeqsm (v.2.3.1; Raumonen *et al*., 2013; Raumonen, 2019), whereby, for each parameter set permutation, five models are generated (Wilkes *et al*., 2023). An optimum model was then selected by minimising the point to cylinder surface distance (Burt *et al*., 2019; Martin‐Ducup *et al*., 2021). Although all trees in MLA‐01 were scanned during the campaign, data were generated for 190 trees, which could be satisfactorily segmented.

Surface area was calculated from terrestrial LiDAR scanning (TLS) using R package treestruct (Shenkin, 2019), which includes the tree bole and all branches captured by the scanner (TLS_t0_). A second stem surface was calculated, which includes the tree bole and all branches captured by the scanner that are larger than 2 cm in diameter, and so branches smaller than 2 cm in diameter were excluded (TLS_t2_). Stem surface area error for both truncations was calculated by using the SD for each tree (based on the surface area estimate of multiple tree QSM per tree) using a Monte Carlo simulation with 10 000 simulations to predict the mean and SE of surface area for the plot.

### Stem surface area estimations

Stem surface area (m^2^) was estimated for all the trees in the plot that also had terrestrial LiDAR data (*n* = 190 trees; Figs S6, S7) and was calculated using an allometric equation based on stem diameter (Chambers *et al*., 2004):

$$\mathrm{SA}=10^{-0.105-0.686X+2.208X^{2}-0.627X^{3}}$$

where SA is the surface area of tree bole and large branches, *X* = log(DBH) in cm which is derived from tree census data (from 2018 to the same year as terrestrial LiDAR data collection). Total surface area from allometric equations (SA_a_) was assigned a SE of 10% (Robertson *et al*., 2010).

A surface area correction coefficient was calculated to adjust the plot stem surface area estimate for the plot height (Robertson *et al*., 2010), thus acknowledging that DBH–height relationships may differ from the Amazonian dataset used in Chambers *et al*. (2004). The correction coefficient is the average ratio of predicted tree height per given DBH within the study plot to trees with the same given DBH in Amazonia. The surface area estimate is then corrected by multiplying the surface area by the correction coefficient. First, a model was created between DBH and height (from forest inventory) for trees in MLA‐01 (*R* ^2^ = 0.77, *P* < 0.001) whereby height = 14.779 × *X*–24.546, whereby *X* = log(DBH in cm). Amazonian tree height was calculated for a plot based in Manaus (Chambers *et al*., 2009) using an equation for East‐Central Amazonia (Feldpausch *et al*., 2011). Average ratio of predicted tree height per given DBH for Amazonian trees to the same diameter in MLA‐01 was estimated at 1.669 (Fig. S8). The estimate surface area is multiplied by the correction coefficient (1.669) to give a height‐adjusted surface area estimate (SA_ha_) with a SE of 10% (Robertson *et al*., 2010).

### Statistical analysis

All statistical analyses were conducted in R (v.4.3.3; R Core Team, 2024). A generalised additive model (GAM), conducted in R package mgcv (Wood, 2011), was fitted to conduct the diel analysis. The model used normalised EA (scaled EA to have a mean of 0 and a SD of 1) as the dependent variable and independent variables included in the model were hour, fitted as a smoother with a cyclic cubic regression spline, and date‐time (cyclic cubic regression spline) nested within tree tag (random effect) as a tensor smoother. An autocorrelation structure was applied to account for temporal dependence in the data, and this was conducted per measurement event as cycles were not continuous. Wilcoxon signed‐rank tests were conducted to test whether predicted values from the model were significantly different from 0.

A mixed‐effects linear model was fitted to determine whether EA varied with tree height, using the R package nlme (Pinheiro *et al*., 2023). The model used EA as the dependent variable, and EA flux measurement height on the stem (m; log transformed) and EA flux measurement position (factor; on the stem, buttress or above first branching point) were independent variables with fixed effects, and tree tag as a random factor (Table S1).

Stem surface area estimates for each tree (*n* = 190) calculated from each method (TLS without truncation, TLS_t0_; TLS with 2‐cm truncation, TLS_t2_; estimated by allometric equations, SA_a_; and using a correction coefficient, SA_ha_) were compared using a Friedman rank sum and a pairwise comparison using a Nemenyi–Wilcoxon–Wilcox test. EA was scaled and stand‐level EA (kg C d^−1^) estimated in accordance with both traditional methods and incorporating vertical variations. First, average EA at 1.1‐m height was scaled to the stem surface area estimates of SA_a_, SA_ha_, TLS_t0_, and TLS_t2_ to conform with more traditional scaling methods to estimate stand‐level EA. Second, the vertical EA model was upscaled, whereby the vertical model was applied to each cylinder of the TLS data for both TLS_t0_ and TLS_t2_ to estimate the stand‐level EA with vertical variations. A mixed‐effects linear model, conducted in the R package lme4 (Bates *et al*., 2015), was used to determine the difference between total EA per tree from each scaling method and the interaction of including vertical variation. Within the model, upscaled EA per tree was the dependent variable and surface area method (SA_a_, SA_ha_, TLS_t0_, and TLS_t2_) and interaction with vertical scaling were the independent variables and tree tag as a random effect.

## Results

### Diel EA

Diel pattern in EA was measured on 18 trees and observed fluxes ranged from 1.13 to 186.7 mg C m^−2^ h^−1^ with a mean EA of 59.30 ± 1.87 mg C m^−2^ h^−1^ across the measurement period (Fig. S9), which is *c*. 1.42 ± 0.05 g C m^−2^ d^−1^. As sampled trees displayed variability in the range of their fluxes (Fig. S9), EA per tree was normalised to remove inter‐tree variability to determine the presence of diel patterns. The GAM showed no significant diel cycle of normalised EA (*P* = 0.051). However, repeating the model with tree tag as a fixed effect showed a significant diel cycle for 16/18 trees studied (Table S2; *R* ^2^ = 0.46; *P* < 0.001). Trees displayed variability within their diel cycles of normalised EA (Fig. S10), which indicates that the bias in measuring at one timestamp is not uniform across trees as the diel cycle is not uniform across the studied trees. Using the latter model, predicted output showed that hours 15:00 h–19:00 h were significantly different from 0 (Table S3); therefore, measurements taken during these hours would not be representative of the mean normalised daily flux. This suggests that measurements taken before 15:00 h, which fall within the usual ‘office hours’, are not significantly different from 0, so measurements taken during these hours would be representative of the mean normalised daily flux at this site (Fig. S10; Table S3). EA data from the diel campaign showed a significant but weak relationship with temperature (temperature log‐transformed; *R* ^2^ = 0.02, *P* < 0.001).

### Vertical EA

**Fig. 1:**
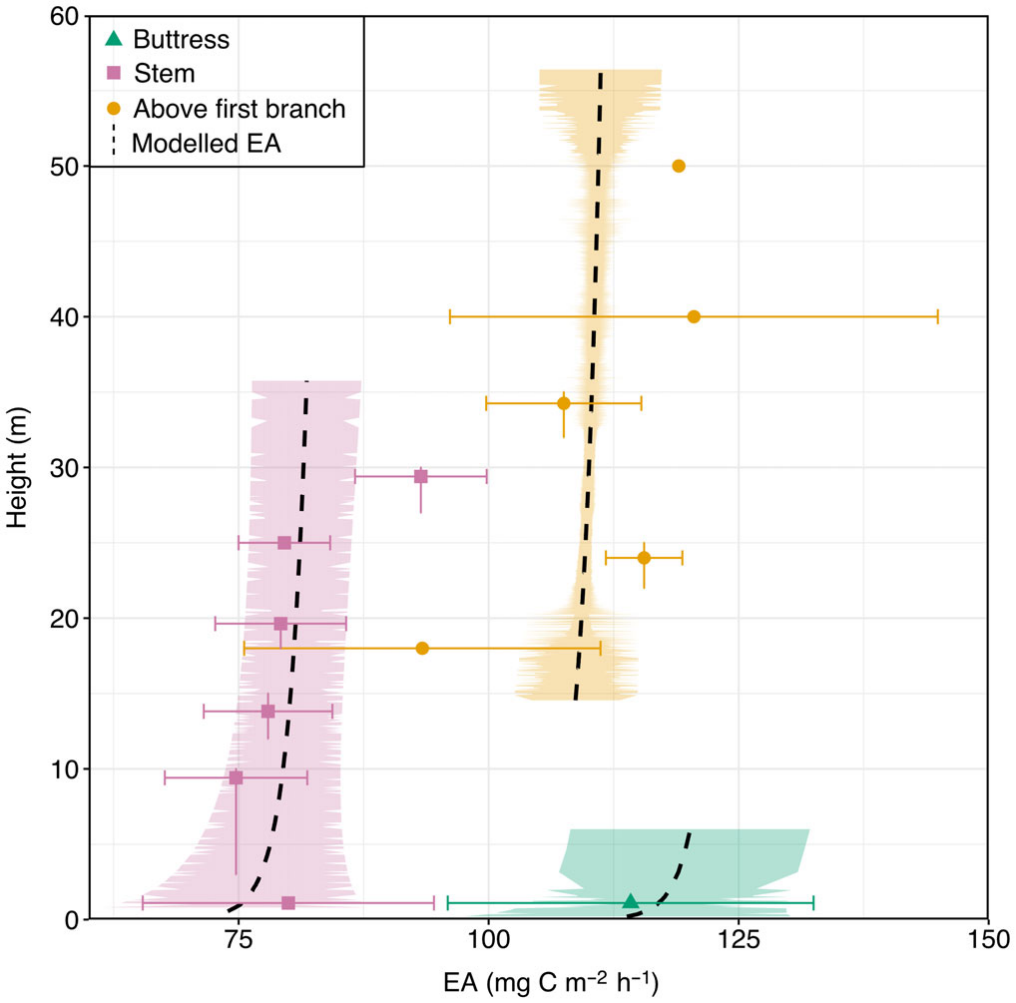
Stem CO_2_ efflux (EA) per unit stem area with tree height (m). Points denote observed values (observations = 68, no. of trees = 13), whereby the average has been calculated in 5 m intervals. Horizontal error bars represent the SE of the mean EA per height interval, and vertical error bars show the range of heights for each interval with the point placed at the average height per interval. The black dashed line represents modelled vertical EA from a mixed‐effects linear model, which was applied to all trees with terrestrial light detection and ranging scanned data and no branch truncation (*n* = 190). Average EA from modelled values was calculated at 0.1‐m intervals, and SE for each interval (represented by bands). For both modelled and observed, green represents measurements on buttresses (triangles), pink represents measurements on the main stem (squares), and orange represents measurements above the first major branching point (circles). Corresponding figure with proportional tree height is available in Supporting Information Fig. S12.

Results showed that EA did not vary with EA measurement height along the stem (log‐transformed measurement height; *P* = 0.61) but did vary on buttresses (*P* < 0.001) and on the stem above the first branching point (*P* < 0.001) (Figs 1, S11, S12; Table S4). Overall, the model had a marginal *R* ^2^ of 0.84 and a conditional *R* ^2^ of 0.25, and the random effect of tree tag was significant (AIC 598 vs 637; Table S1). Neither stem diameter (*P* = 0.48) nor its interaction with flux measurement height (*P* = 0.23) were significant (Table S1).

### Stem surface area estimates

**Fig. 2:**
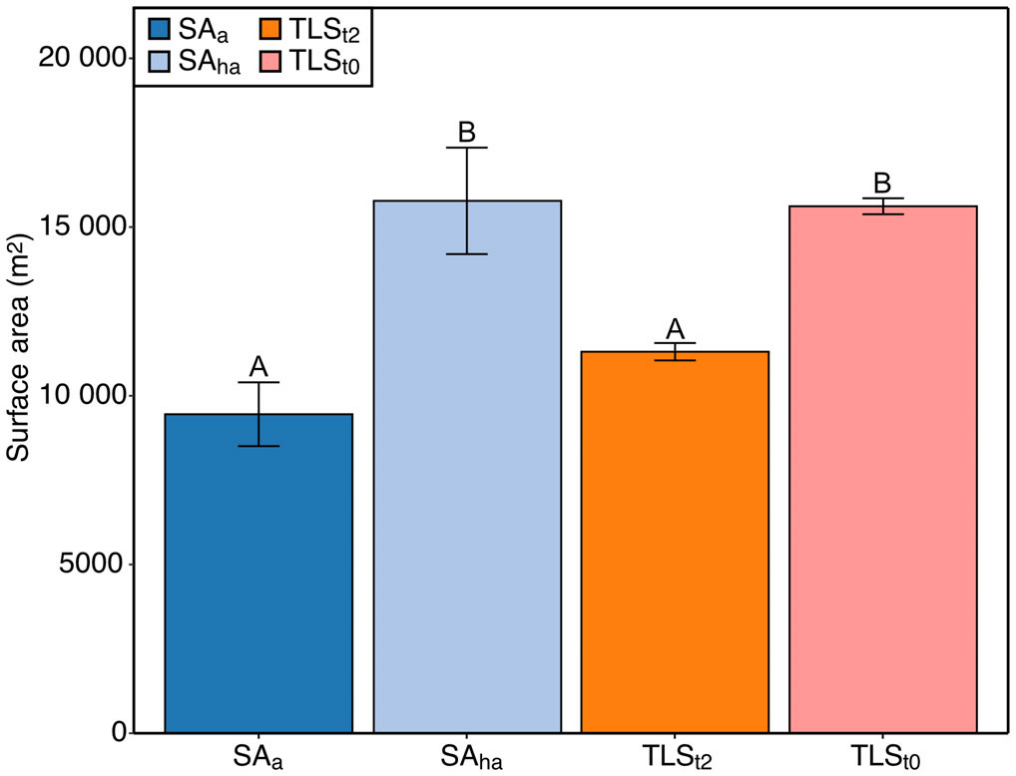
Stem surface area (m^2^) of study trees (*n* = 190) as calculated by the allometric equation from Chambers *et al*. (2004) (SA_a_; dark blue), using the allometric equation with a plot‐specific height adjustment coefficient applied (SA_ha_; light blue), terrestrial light detection and ranging (LiDAR) scanning with 2‐cm truncation (TLS_t2_; orange) and terrestrial LiDAR scanning with no truncation (TLS_t0_; light pink). Error bars represent SE, whereby SA_a_ and SA_ha_ errors have been assigned at 10% (Robertson *et al*., 2010) and the dashed line indicates the traditional stem surface area estimate from SA_a_. Letters denote statistical significance (*P* < 0.05), as determined by Friedman rank sum and a pairwise comparison using a Nemenyi–Wilcoxon–Wilcox test, whereby different letters denote significant difference between groups, and shared letters show no significant difference between groups. The results are given in Supporting Information Table S5.

Stem surface area (m^2^) was estimated for all the trees in the plot that were also scanned during the terrestrial LiDAR campaign, which was a total of 190 trees (Figs 2, S6, S7; Table S5). There was a significant difference in stem surface area from each method overall (chi‐squared = 242.13; df = 3; *P* = <0.001). Specifically, there was a significant difference between SA_a_ and SA_ha_ (*P* < 0.001), SA_a_ and TLS_t0_ (*P* < 0.001), SA_ha_ and TLS_t2_ (*P* < 0.001), and TLS_t2_ and TLS_t0_ (*P* < 0.001). There was no difference between SA_a_ and TLS_t2_ (*P* = 0.38) and between SA_ha_ and TLS_t0_ (*P* = 0.38) (Fig. 2).

### Upscaling EA to stand‐level

**Fig. 3:**
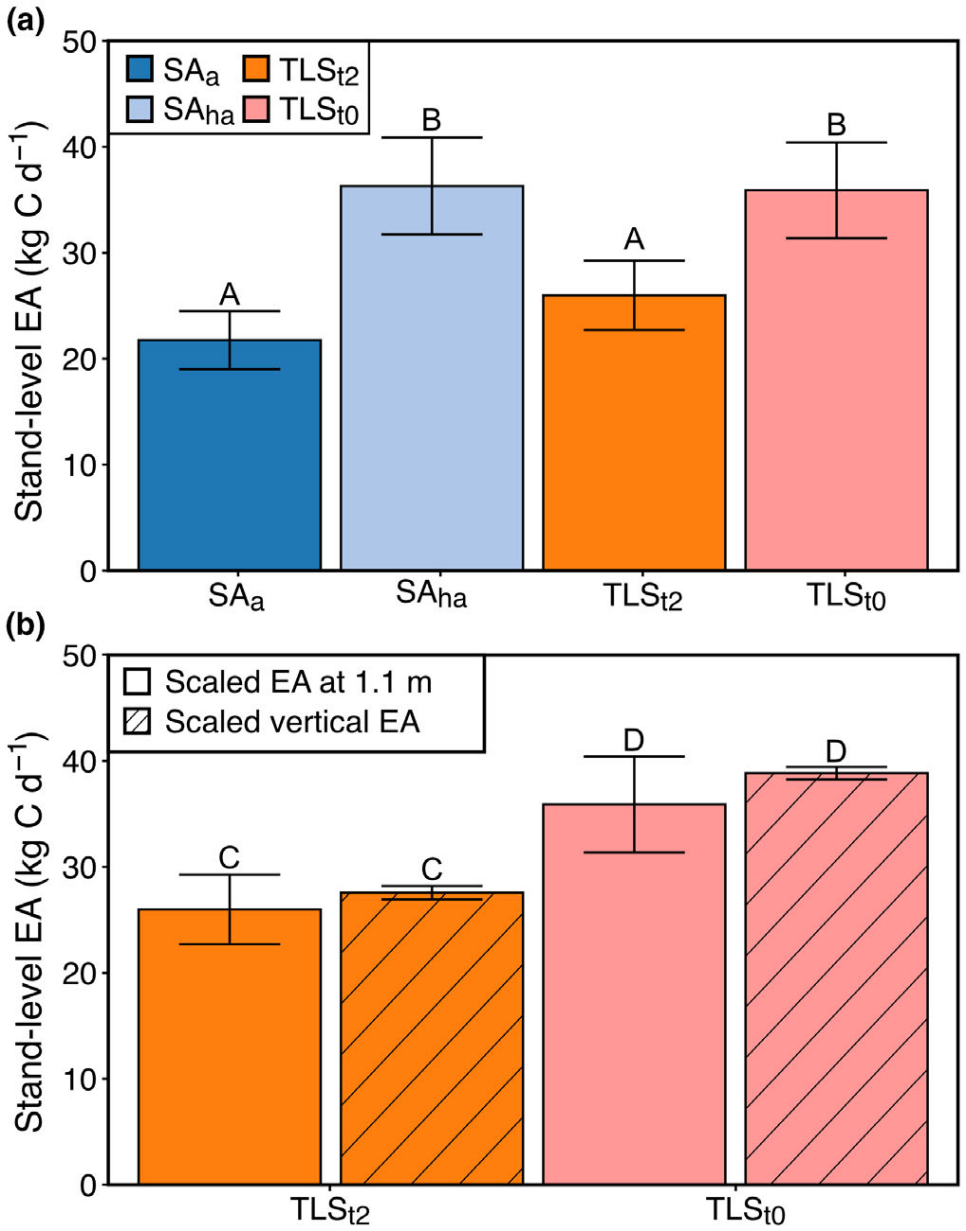
Upscaled stem CO_2_ efflux (EA) by each stem surface area estimation method and with vertical EA variation. (a) shows stand‐level EA determined by average EA at 1.1 m (95.77 ± 12.07 mg C m^−2^ h^−1^) scaled to surface area estimated from the allometric equation from Chambers *et al*. (2004) (SA_a_; dark blue), using the allometric equation with a site‐specific correction coefficient applied (SA_ha_; light blue), terrestrial light detection and ranging (LiDAR) scanning with 2‐cm truncation (TLS_t2_; orange) and terrestrial LiDAR scanning with no truncation (TLS_t0_; light pink) with SE. (b) shows stand‐level EA calculated with (hatched) and without (solid) vertical variation included for TLS_t2_ (orange) and TLS_t0_ (pink) with SE. Letters indicate significant differences (*P* < 0.05), as determined by a mixed‐effects linear model, whereby different letters denote significant differences between groups, and shared letters show no significant difference between groups. The results are given in Supporting Information Tables S6 and S7.

Stand‐level EA was estimated (kg C d^−1^) for the plot following traditional methodologies and including vertical variation. Average EA at 1.1 m (95.77 ± 12.07 mg C m^−2^ h^−1^) was scaled to surface area estimates in line with traditional methodologies, and the vertical EA model was applied to the terrestrial LiDAR data for TLS_t0_ and TLS_t2_ (Fig. 3a; Table S6). The model showed that the surface area estimation method was the primary driver for stand‐level EA and that vertical variation had no significant effect on stand‐level EA estimates. Specifically, the results showed that when upscaling EA at 1.1 m, there was a significant difference between stand‐level EA from SA_a_ and SA_ha_ (*P* < 0.001), SA_a_ and TLS_t0_ (*P* < 0.001), SA_ha_ and TLS_t2_ (*P* < 0.001), and TLS_t0_ and TLS_t2_ (*P* < 0.001), and nor was any significant difference between SA_a_ and TLS_t2_ (*P* = 0.082), and SA_ha_ and TLS_t0_ (*P* = 0.99) (Fig. 3a; Table S7). This suggested that not correcting surface area from SA_a_ to SA_ha_ would lead to a *c*. 40% underestimation in stand‐level EA. When considering the inclusion and exclusion of vertical variation, results showed no significant difference between TLS_t2_ including and excluding vertical variation in EA (*P* = 0.92) and TLS_t0_ including and excluding vertical variation in EA (*P* = 0.43) (Fig. 3b; Table S7).

## Discussion

### Diel EA

This study aimed to quantify the biases in measuring EA in the field and the implications for scaling EA to stand‐level with the intention of providing recommendations to mitigate these biases. This study found no significant uniform diel cycle in EA across trees, but significant inter‐tree variability and individual diel patterns of EA, with some trees displaying afternoon peaks in EA and others displaying afternoon depressions (Fig. S10). Previous research has shown higher EA during the night decreasing in the early morning, likely following ascending xylem sap and elevated air temperatures in the morning (Kunert, 2018) and reduced cell turgor and, therefore, growth at night (Salomón *et al*., 2018; Zweifel *et al*., 2021). EA daytime suppressions have previously been associated with elevated crown temperature, vapor pressure deficit (VPD), and high transpiration rates (Jardine *et al*., 2022). In this study, however, the relationship between EA and temperature displayed a weak positive correlation (*R* ^2^ = 0.02, *P* < 0.001). As research conducted outside the tropics evidences strong thermal patterns in stem respiration (Stockfors, 2000; Zha *et al*., 2004; Saveyn *et al*., 2008), this may suggest that the lack of a uniform pattern observed at this site is due to the limited temperature range over the study period (22.4–30.9°C; Fig. S3). Similar to a study undertaken elsewhere in Malaysian Borneo, there was no clear effect of environmental factors on diel variation of EA, and different trees displayed different patterns in diel EA (Katayama *et al*., 2016), indicating that the bias in measuring at one timestamp is not uniform across trees (Figs S9, S10). Nevertheless, given the trade‐off between the logistical constraints of measuring diel variations in EA and the potential bias in not accounting for such variations, it is important to provide a guideline for when to measure EA. Although the overall model showed no diel cycle, when considering the individual diel cycles of studied trees, results suggested that measuring before 15:00 would be a suitable time frame to measure EA, which fortuitously coincides with typical working hours. This research has, however, only been conducted in one geographic location within an old‐growth forest and so is limited in scope. Nevertheless, it could be used as guidance within this region. Ideally, a subset of trees would be monitored with continuous, automatic systems to capture diel variation, whilst the wider spatial sampling would take place at the most representative time of the day.

### Vertical EA

This study also aimed to determine the bias of measuring EA at breast height only and quantified vertical variations in EA. Results showed that EA variation was not related to measurement height or diameter, but to position on the stem, and higher EA was observed on buttresses and on the stem above the first branching point. Research has shown that dissolved CO_2_ in the sap flow is transported upwards in the morning and is diffused out through the thinner bark in the canopy (Teskey & Mcguire, 2002), which would result in lower EA at breast height relative to the canopy (Katayama *et al*., 2014). Similarly, CO_2_ originating in the root system may be transported upwards and diffuse out in the xylem, as physiological buttresses are the result of a secondary xylem formed on the upper side of the lateral roots (Nölke *et al*., 2015), resulting in enlarged and thickened roots at the tree base to aid mechanical support (Simpson, 2010). However, consistently higher EA above the branching points and on buttresses (Fig. S11) was observed despite data being collected across the daytime hours (09:00 h–15:30 h), and likely not the result of increased diffusion of CO_2_ transported by sap flow. This suggests that different physiological conditions are responsible for this elevated EA on buttresses and above the first branching point.

Higher EA on buttresses and above branching points may be due to variations in stem growth (Araki *et al*., 2015), which would indicate a stable growth rate across the main stem, explaining why no variation in EA was observed on tree stems in this study. Although all measurements were taken on the main stem (or on the first‐order branch if the tree had a Y‐shaped architecture), higher EA above the first branch is consistent with a higher basal respiration rate for branches than stems (Damesin *et al*., 2002) and with previously observed higher CO_2_ efflux rate of wood in the upper canopy than the lower canopy of stems of the same given diameter (Cavaleri *et al*., 2006). Higher EA above the first branching point may be the result of the metabolic cost of sugar and water transfer in the canopy. Woody respiration is thought to increase higher up in the canopy closer to leaves where there is an increased energy cost of growing new cells and the exchange of carbohydrates into and out of the phloem from the xylem parenchyma cells (Sprugel, 1990). Research on wood nutrient content in Sabah found that bark nutrient content was higher in branches, likely due to branches having a higher proportion of rich inner bark than trunks and stems (Inagawa *et al*., 2023). This same study also observed lower concentrations of wood nutrients in the trunk vs higher concentrations in coarse roots and branches (Inagawa *et al*., 2023). Research into wood anatomical and hydraulic properties of roots, stems, and branches in Indonesia showed that vessels tended to be larger in the main stem and smaller in coarse roots and branches (Kotowska *et al*., 2015). Similar results have also been observed in South America where the largest vessels were within the stem (Machado *et al*., 2007) and branches and roots displayed similar vessel sizes (Fortunel *et al*., 2014). It has been suggested that these differences could represent a response to permanent water availability and low evaporative demand (Kotowska *et al*., 2015). Such differences in nutrient and vessel traits between branches, buttresses (coarse roots), and stems indicate further physiological differences and possible mechanisms that may be responsible for the patterns of EA observed in this study.

Although there is currently limited research on the woody CO_2_ efflux of buttresses, making it difficult to determine mechanisms for the observed elevated EA on buttresses, research into coarse root respiration has found a relationship with diameter (Makita *et al*., 2012). However, when considering just EA on buttresses, we found no relationship between diameter and EA (linear model; *P* = 0.47). Buttress growth rate could also be responsible for the higher EA on buttresses, although this would be difficult to determine given standard practice involves measuring diameter 50 cm above the top of the buttress at the tree census (Marthews *et al*., 2014), although it is possible to correct for this (Cushman *et al*., 2014). Buttresses have been found to have decreased vessel size, vessel area and specific conductivity from the core to outer wood in accordance with increased mechanical loading (Christensen‐Dalsgaard *et al*., 2008) and reportedly have thinner bark (Barlow *et al*., 2003). It is potentially these anatomical differences, stress from the increased mechanical loading, and resulting differences in metabolism in the buttresses that are responsible for the increased EA observed within this study. Overall, the model did support these observed patterns of elevated EA on buttresses and above branching points (Fig. S11) across the range of tree sizes and species sampled, but further research is needed to disentangle the mechanisms behind this elevated EA. This research was limited by being unable to measure branch EA due to the size of the chamber employed, which should be a focal point of future research.

### Upscaling EA to stand‐level

Understanding the implications of accounting for vertical variations in EA relies on having accurate estimates of stem surface area. When scaling vertical variation in EA to stand‐level, results showed that scaling the average EA at 1.1 m to SA resulted in an underestimation of stand‐level EA by *c*. 40% compared with TLS_t0_ (without vertical variation). However, when scaling using a surface area correction coefficient (SA_ha_), there was no difference between using terrestrial LiDAR and using a correction coefficient, and no difference was found if vertical variation was or was not accounted for. Here, vertical variation in EA does not impact stand‐level EA estimates if stem surface area is correctly estimated, indicating that measuring at 1.1 m is adequate for EA methodologies. Such findings support research elsewhere in Malaysia that whole‐tree EA estimates, including vertical measurements, did not differ from those based solely on breast height measurements (Katayama *et al*., 2014). This was suggested to be because of the reduced stem surface area in the canopy where EA was elevated and so resulted in little effect of vertical variation on whole‐tree estimates (Katayama *et al*., 2014). Additionally, although buttresses had a higher EA than stems, these results show that having some trees with buttresses in the sample will not impact the overall stand‐level EA (6/13 sampled trees with buttresses; 66/397 of 1‐ha plot with buttresses). Overall, these findings suggest that the uncertainties in the stem surface area estimates appear to be more important than vertical variation for the accuracy of stand‐level EA estimations. As there was no difference in stem surface area estimate from TLS_t0_ and SA_ha_, this highlights the importance of applying a site‐specific correction factor to account for the biogeographic differences in stem diameter–height relationships (Feldpausch *et al*., 2011; Meir *et al*., 2017). Although there was no difference in using a correction coefficient and using terrestrial LiDAR, future research should continue to employ terrestrial LiDAR to validate the performance of site‐specific correction factors across forest types and regions.

Results from this study show that underestimation of stand‐level EA due to inaccuracy from employing allometric equations would have consequences for the forest carbon budget. For the 190 trees investigated, traditional estimates of stand‐level EA (SA_a_) resulted in *c*. 40% underestimation compared with using TLS_t0_ and SA_ha_. Such underestimation would have implications for the stand‐level estimates of autotrophic respiration (*R*_a_), ecosystem respiration (*R*_eco_), and gross primary productivity (GPP) if GPP is quantified using bottom‐up methods, whereby GPP = net primary productivity (NPP) + *R*_a_ (Malhi *et al*., 2014; Mills *et al*., 2023). Based on this study, GPP, *R*_a_, and *R*_eco_ for this forest may be larger than previously estimated. The estimate for the net carbon balance, on the contrary, would not be affected if calculated as *R*_eco_ – GPP, as EA is a component of *R*_a_, which is included in both terms, and any changes would thus cancel out.

In conclusion, measuring EA accurately can be logistically difficult and involves labour‐intensive fieldwork. Although results from this study are restricted to one geographic region, our outcomes indicate that logistical constraints can be overcome by measuring EA in the correct time frame and creating and applying a site‐specific stem surface area correction coefficient. This is necessary where TLS is unavailable to account for the local diameter–height relationship and to improve the accuracy of EA estimates. Results from this study showed that EA does vary along the stem, but vertical variations do not impact stand‐level EA when stem surface area is accurately estimated and so measuring at 1.1 m would suffice. Conclusions from this case study can be used as a guideline for future research and experimental design, as it is acknowledged that the study was limited in both space and time.

## Competing interests

None declared.

## Author contributions

MBM wrote the paper with contributions from TR, AS, PW, MD, SP, JCB and JK. MBM analyzed the data. YM, AS, PW, MD, RR and RN contributed new reagents/analytic tools. MBM performed the research. MBM and TR designed the research.

## Disclaimer

The New Phytologist Foundation remains neutral with regard to jurisdictional claims in maps and in any institutional affiliations.

## Supporting information


**Fig. S1** Occupied basal area (%) by Genus of the trees sampled and of the 1‐ha study plot.
**Fig. S2** Occupied basal area (%) by diameter class of the trees sampled and of the 1‐ha study plot.
**Fig. S3** Diurnal temperature (°C) and relative humidity (%) over the study period.
**Fig. S4** Diagram of set‐up and photographs of diel stem CO_2_ efflux campaign.
**Fig. S5** Photographs of set‐up for vertical stem CO_2_ efflux campaign.
**Fig. S6** Total woody stem surface area (m^2^) per tree calculated by SA_a_ and TLS_t0_.
**Fig. S7** Total woody stem surface area (m^2^) per tree calculated by SA_ha_ and TLS_t0_.
**Fig. S8** Tree height–diameter curve for trees within the study plot.
**Fig. S9** Observed diel stem CO_2_ efflux.
**Fig. S10** Modelled normalised diel stem CO_2_ efflux for study trees as fixed effect.
**Fig. S11** Boxplot of stem CO_2_ efflux observation on buttresses, stems, and above first branch.
**Fig. S12** Modelled and observed stem CO_2_ efflux with proportional height (%).
**Table S1** Models and Akaike information criterion created for vertical stem CO_2_ efflux model.
**Table S2** Diel normalised stem CO_2_ efflux model generalised additive model output.
**Table S3** Diel normalised stem CO_2_ efflux Wilcoxon output.
**Table S4** Model coefficients for vertical stem CO_2_ efflux model.
**Table S5** Stand‐level stem surface area.
**Table S6** Stand‐level stem CO_2_ efflux values upscaled with and without vertical variation.
**Table S7** Model output stand‐level stem CO_2_ efflux upscaled with and without vertical variation.
Please note: Wiley is not responsible for the content or functionality of any Supporting Information supplied by the authors. Any queries (other than missing material) should be directed to the *New Phytologist* Central Office.

## Data Availability

Dataset of diel and vertical stem CO_2_ efflux is available at doi: 10.5281/zenodo.14408902 and data set of terrestrial LiDAR scans for MLA‐01 is available at doi: 10.5285/844bd5c5bc9940d6b04cd35bd9c8b956.
