# Supplementary material for: Investigating the accuracy of tropical woody stem CO2 efflux estimates: scaling methods, and vertical and diel variation
Source: New Phytol. 2025 Apr 3;246(5):2004–14. doi: 10.1111/nph.70122 (PMC12059531; doi:10.1111/nph.70122)
Supplement: Supplementary file 1 — Fig. S1 Occupied basal area (%) by Genus of the trees sampled and of the 1‐ha study plot. Fig. S2 Occupied basal area (%) by diameter class of the trees sampled and of the 1‐ha study plot. Fig. S3 Diurnal temperature (°C) and relative humidity (%) over the study period. Fig. S4 Diagram of set‐up and photographs of diel stem CO2 efflux campaign. Fig. S5 Photographs of set‐up for vertical stem CO2 efflux campaign. Fig. S6 Total woody stem surface area (m2) per tree calculated by SAa and TLSt0. Fig. S7 Total woody stem surface area (m2) per tree calculated by SAha and TLSt0. Fig. S8 Tree height–diameter curve for trees within the study plot. Fig. S9 Observed diel stem CO2 efflux. Fig. S10 Modelled normalised diel stem CO2 efflux for study trees as fixed effect. Fig. S11 Boxplot of stem CO2 efflux observation on buttresses, stems, and above first branch. Fig. S12 Modelled and observed stem CO2 efflux with proportional height (%). Table S1 Models and Akaike information criterion created for vertical stem CO2 efflux model. Table S2 Diel normalised stem CO2 efflux model generalised additive model output. Table S3 Diel normalised stem CO2 efflux Wilcoxon output. Table S4 Model coefficients for vertical stem CO2 efflux model. Table S5 Stand‐level stem surface area. Table S6 Stand‐level stem CO2 efflux values upscaled with and without vertical variation. Table S7 Model output stand‐level stem CO2 efflux upscaled with and without vertical variation. Please note: Wiley is not responsible for the content or functionality of any Supporting Information supplied by the authors. Any queries (other than missing material) should be directed to the New Phytologist Central Office. [file NPH-246-2004-s001.pdf]

New Phytologist

***New Phytologist* Supporting Information**

Article title: Investigating the accuracy of tropical woody stem CO_2 efflux estimates: scaling methods, and vertical and diel variation.

Authors: Maria B. Mills, Alexander Shenkin, Phil Wilkes, Mathias Disney, Susan Page, Juan Carlos Berrio, Jörg Kaduk, Yadvinder Malhi, Rolando Robert, Reuben Nilus & Terhi Riutta

Article acceptance date: 12 March 2025

The following Supporting Information is available for this article:

Figure S1: Occupied basal area (%) by Genus of the trees sampled and of the 1-ha study plot.
Figure S2: Occupied basal area (%) by diameter class of the trees sampled and of the 1-ha study plot.
Figure S3: Diel temperature (°C) and relative humidity (%) over the study period.
Figure S4: Diagram of set up and photographs of diel stem CO_2 efflux campaign.
Figure S5: Photographs of set up for vertical stem CO_2 efflux campaign.
Figure S6: Total woody stem surface area (m^2) per tree calculated by SA_a and TLS_{t0}.
Figure S7: Total woody stem surface area (m^2) per tree calculated by SA_{ha} and TLS_{t0}.
Figure S8: Tree height-diameter curve for trees within the study plot.
Figure S9: Observed diel stem CO_2 efflux.
Figure S10: Modelled normalized diel EA for study trees as fixed effect.
Figure S11: Boxplot of stem CO_2 efflux observation on buttresses, stems and above first branch.
Figure S12: Modelled and observed stem CO_2 efflux with proportional height (%)

Table S1: Models and Akaike information criterion created for vertical stem CO_2 efflux model.
Table S2: Diel normalized EA model GAM output
Table S3: Diel normalized EA Wilcoxon output
Table S4: Model coefficients for vertical stem CO_2 efflux model.
Table S5: Stand-level stem surface area
Table S6: Stand-level EA values upscaled with and without vertical variation
Table S7: Model output Stand-level EA upscaled with and without vertical variation

Fig. S1 Occupied basal area (%) by Genus in the 1-ha study plot (A) and number of trees sampled in the vertical and diel campaign (B). Colours represent different genera. Only genus contributing > 1% of basal area were included in A. The most common family sampled was Dipterocarpaceae (50 % in diell; 47 % in vertical), however, Dipterocarpaceae makes up ~62 % of tree basal area within this 1-ha plot.

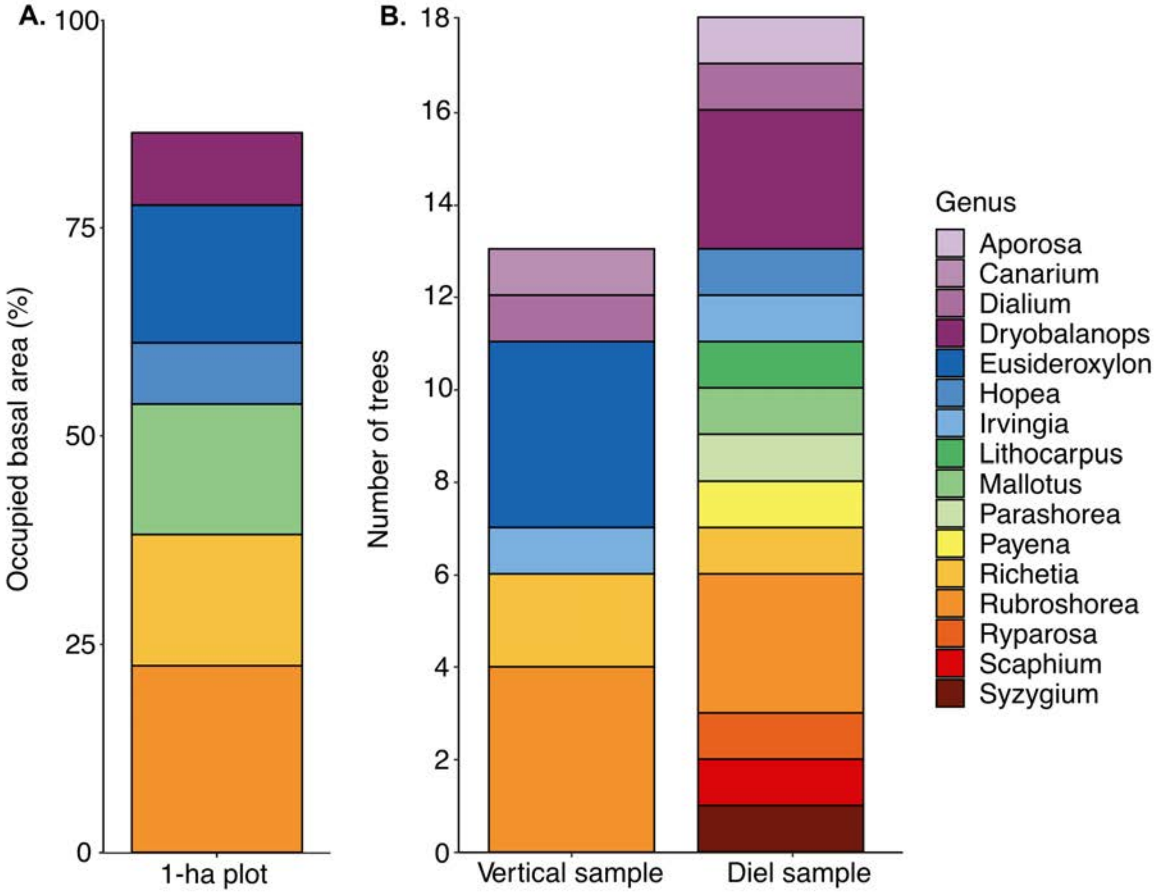

Fig. S2: Occupied basal area (%) by diameter class (diameter at 1.3 m height) in the 1-ha study plot (A) and of the trees sampled in the vertical and diel campaign (B). Colours represent different diameter classes and the number of trees in each class is shown in each bar in A.

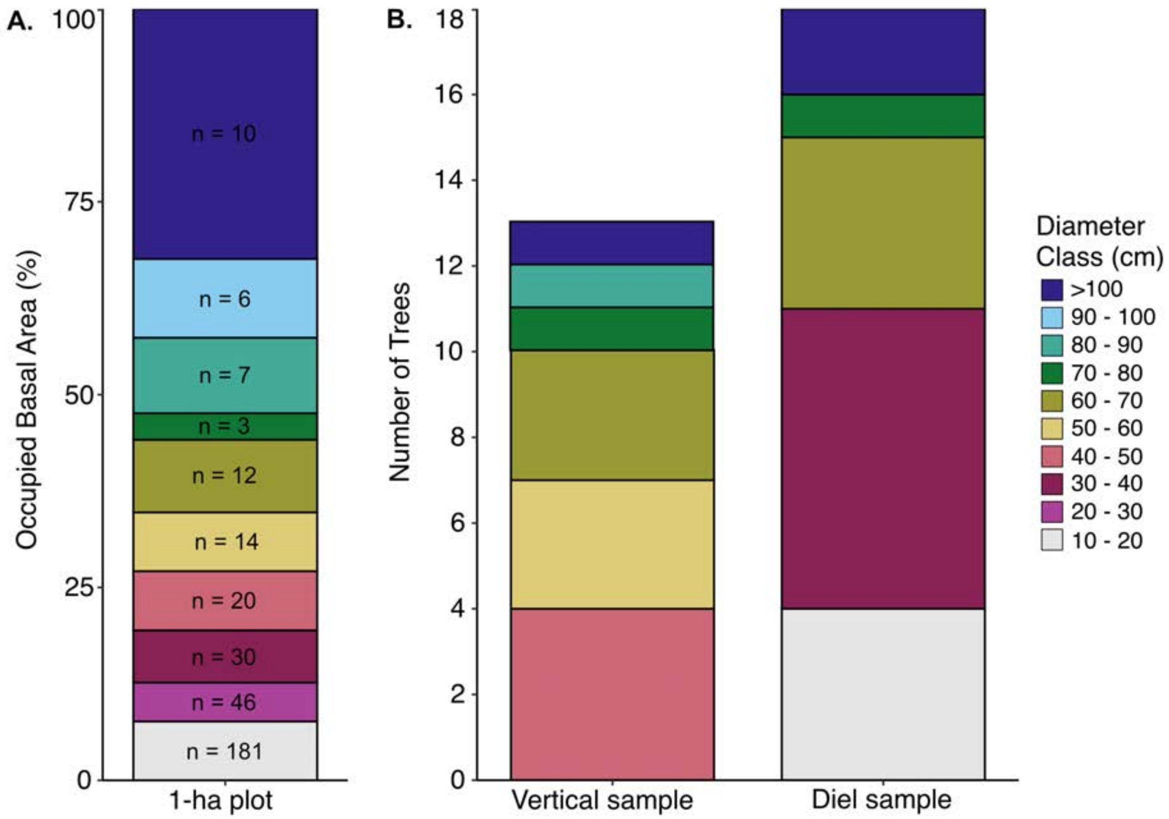

Fig. S3: Diel temperature (°C; A) and relative humidity (%; B) over the nine-day study period. Grey dots represent observations (every 30 minutes in four locations across the 1-ha plot), and black line represents trend as determined by a Generalised Additive Model (GAM), with temperature ($R^2 = 0.58$, edf = 7.88, F = 172.94, p <0.001) and relative humidity ($R^2 = 0.25$, edf = 7.91, F = 41.07, p <0.001) as function of time with Tinytag sensor as a random factor. Blue bands represent 95 % confidence interval.

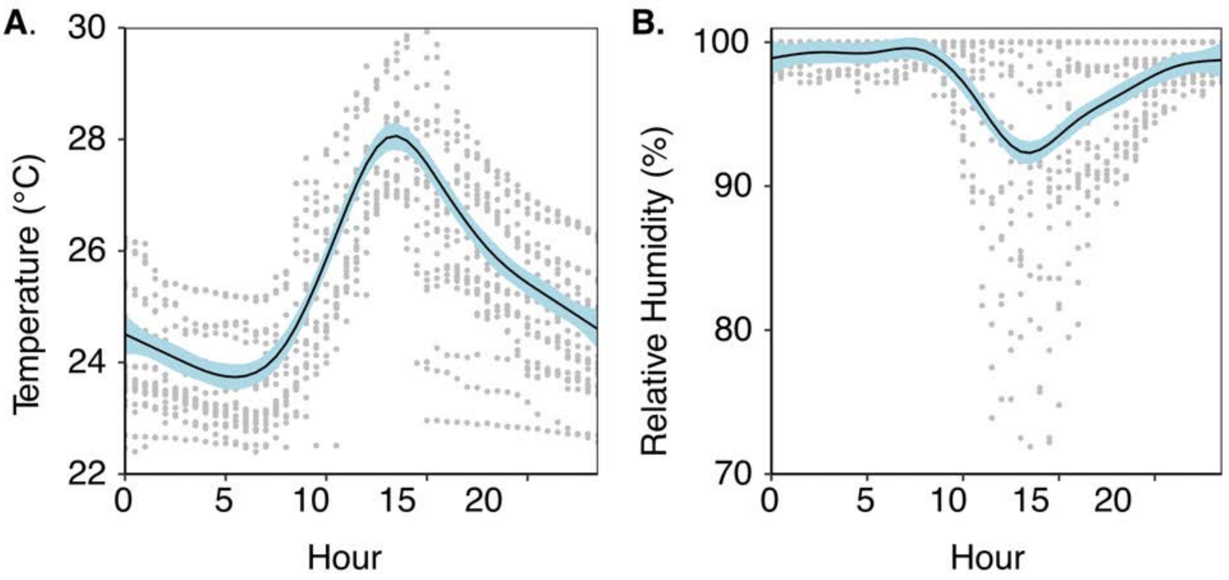

Fig. S4: Diagram of set up and photographs of diel stem CO_2 efflux campaign. Diagram is not to scale. Photographs are authors own

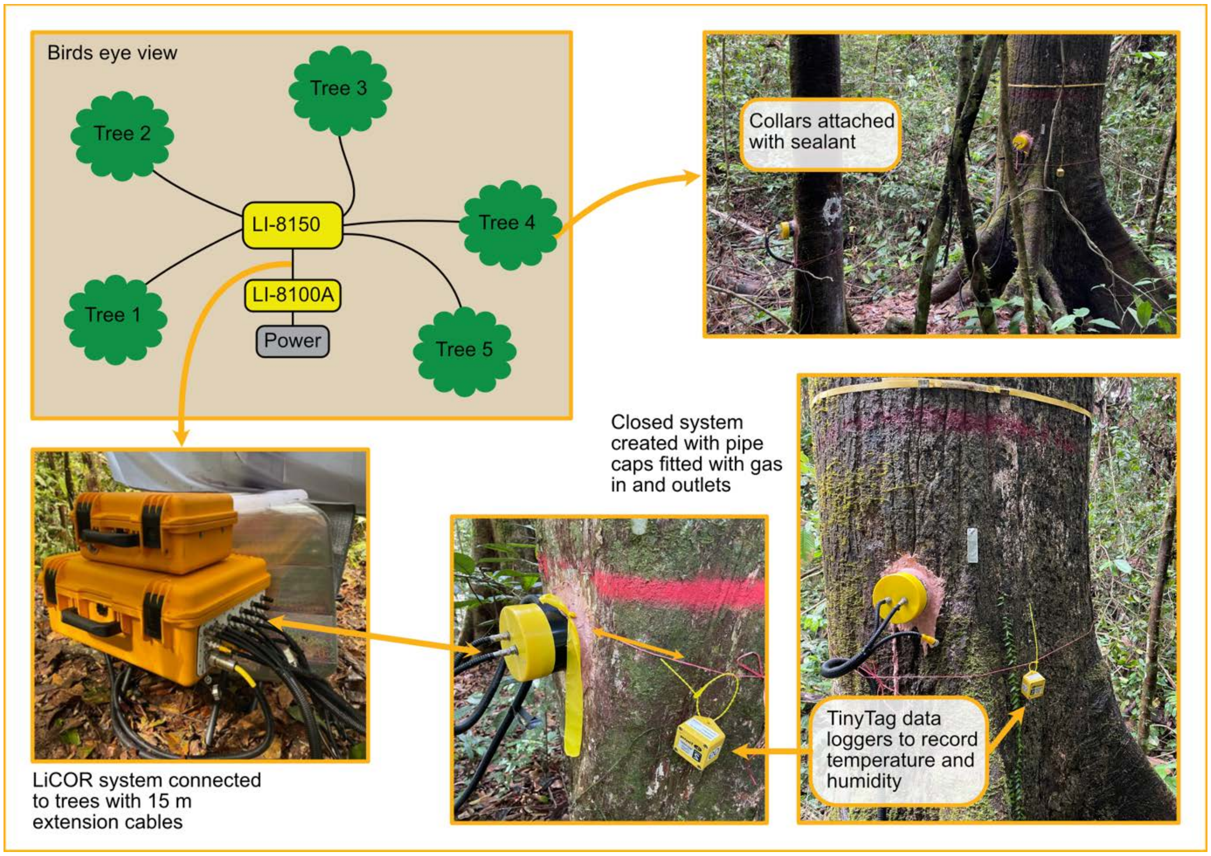

.

Fig S5: Photographs of set up for vertical stem CO_2 efflux campaign. Photographs are authors own, except for the bottom right view of the climber, of which belongs to Unding Jami.

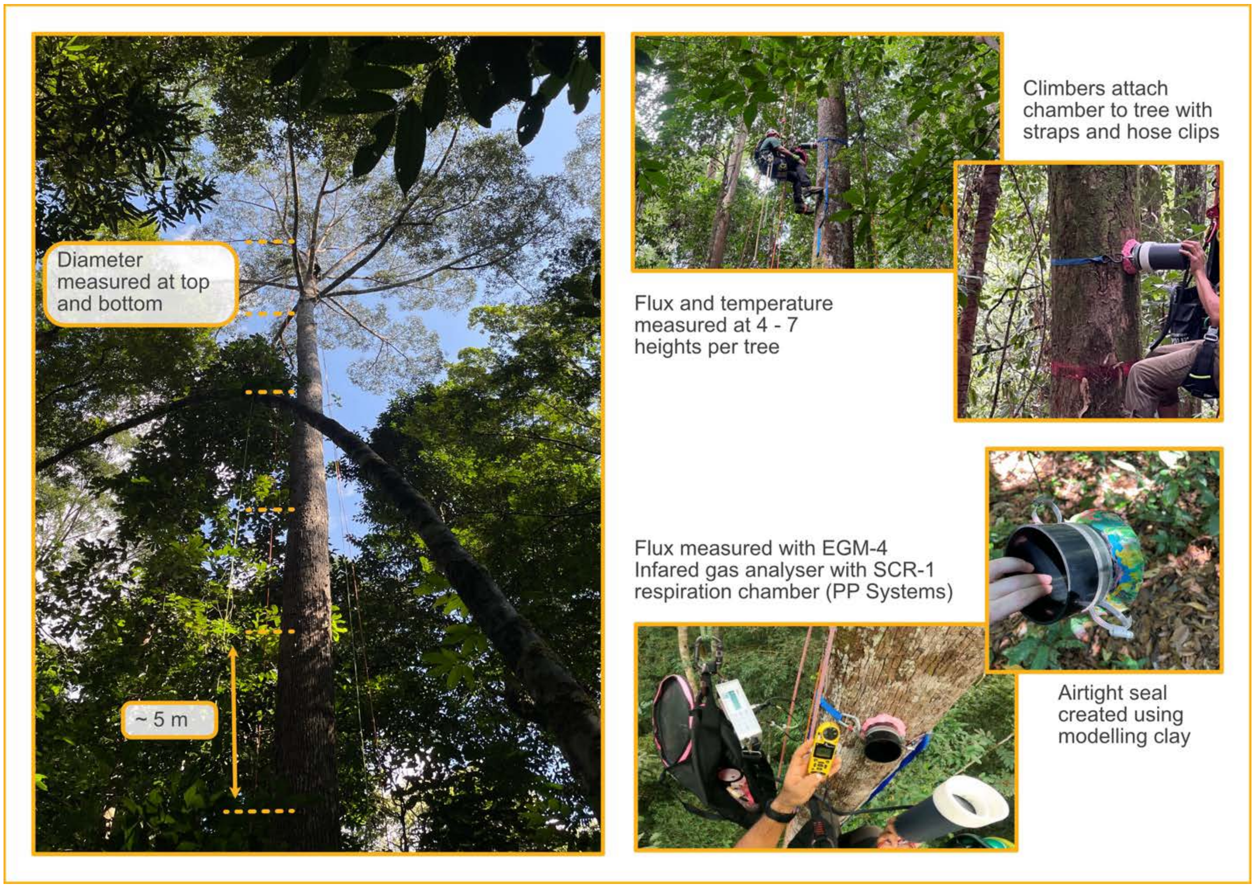

New Phytologist

Fig. S6: Total woody stem surface area (m^2) per tree tag at the study site, calculated by from an allometric equation based on tree diameter (Chambers *et al.*, 2004) ($SA_{a;}$ dark blue) and from terrestrial LiDAR scanning with no truncation (TLS; pink). Only trees scanned in the terrestrial LiDAR campaign were included, which was 190 trees.

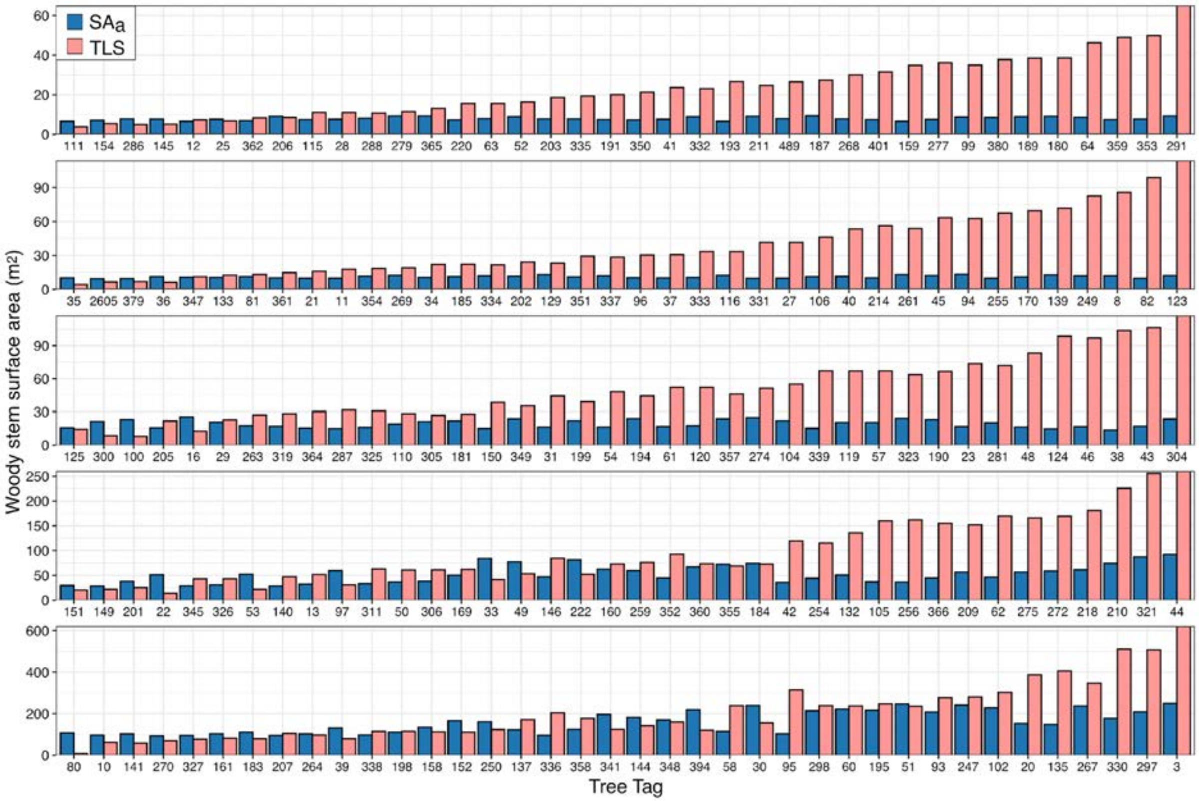

Fig. S7: Total woody stem surface area (m^2) per tree tag at the study site, calculated by from an allometric equation based on tree diameter (Chambers *et al.*, 2004) with a site-specific correction-coefficient ($SA_{ha;}$ blue) and from terrestrial LiDAR scanning with no truncation (TLS; pink). Only trees scanned in the terrestrial LiDAR campaign were included, which was 190 trees.

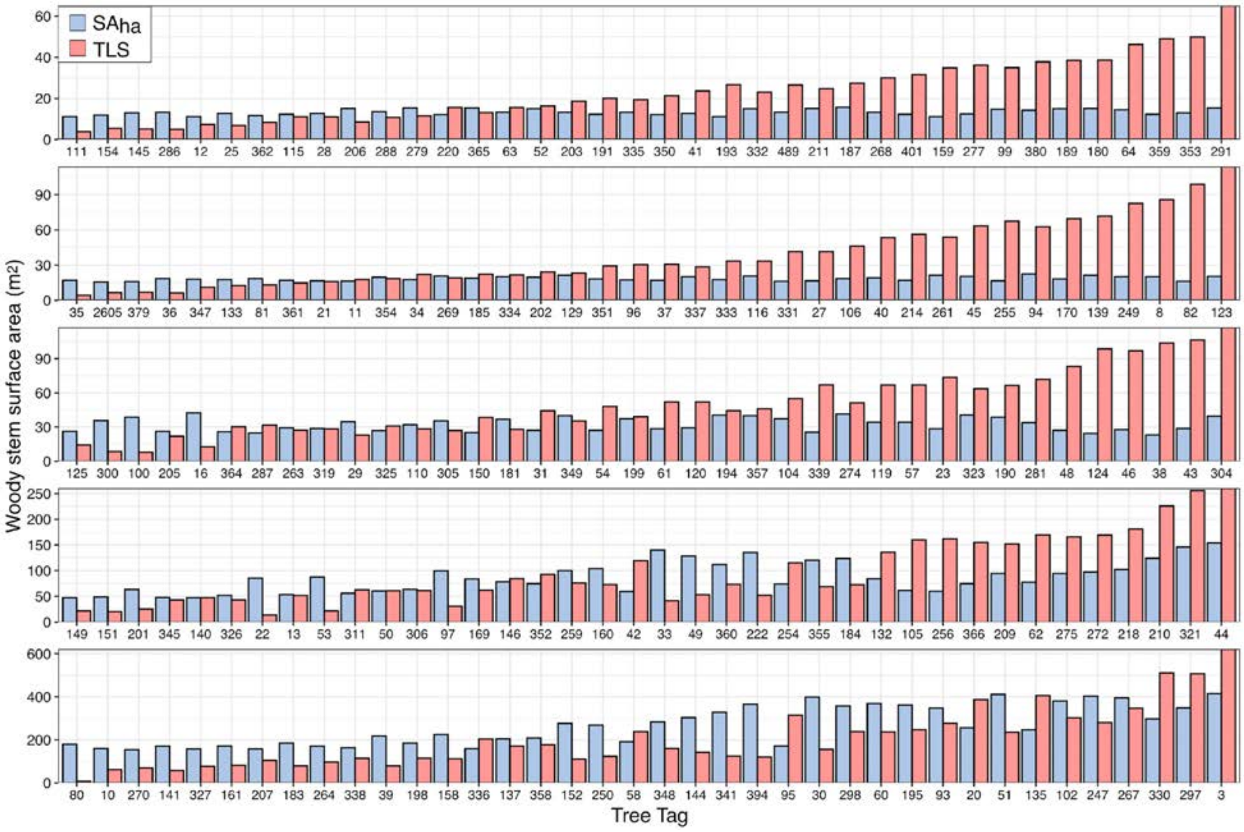

Fig. S8: Relationship between tree height (m) and tree diameter at 1.3 m (DBH), both derived from forest inventory, for trees in the study plot whereby black points represent observations. Green line denotes the relationship between height and DBH for this plot, whereby height = 14.779*X - 24.546, and X = log(DBH) in cm. Blue dashed line represents height curve of trees in East-Central Amazonia calculated from a regional-classification-structure equation (Feldpausch et al., 2011) whereby log(height) = 0.93633 +0.4982X + 0.0109, and X = log(DBH) applied to a plot in Manaus (Chambers *et al.,* 2009).

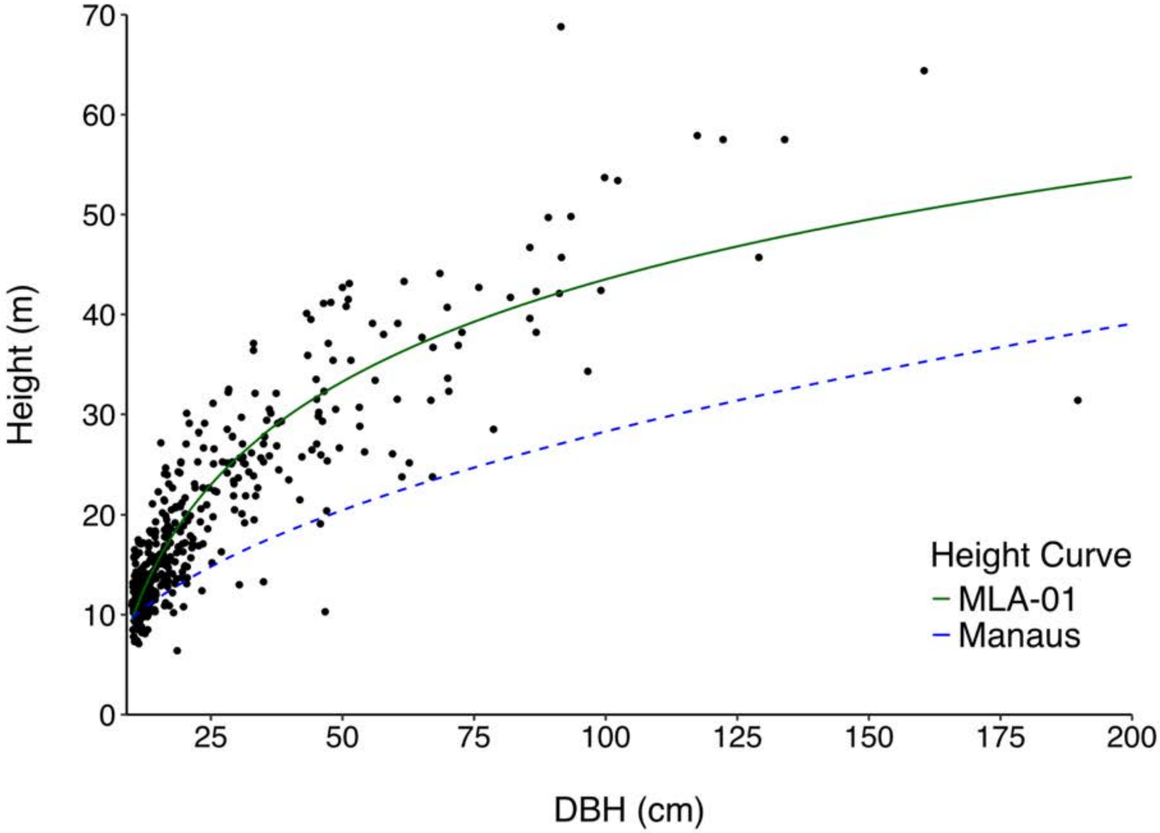

Figure S9: Woody stem CO_2 efflux (EA) per unit stem area of trees sampled in the diel campaign. Points are average values per hour per tree (denoted by differing colours). Trees within the same group were measured simultaneously - the measurement system was moved from one group to the next after 48 to 72 hours of measurements. A generalised additive model (GAM) was fitted using R package "mgcv" (Wood, 2011), whereby hour was cyclic cubic regression spline (edf = 2.12, F = 28.98, p = 0.006) and tree tag was a random effect (edf = 16.97, F = 511.31, p <0.001) which had an adjusted R^2 = 0.93. Black line shows predicted fit at population level for the GAM model and 95 % confidence interval by grey bands.

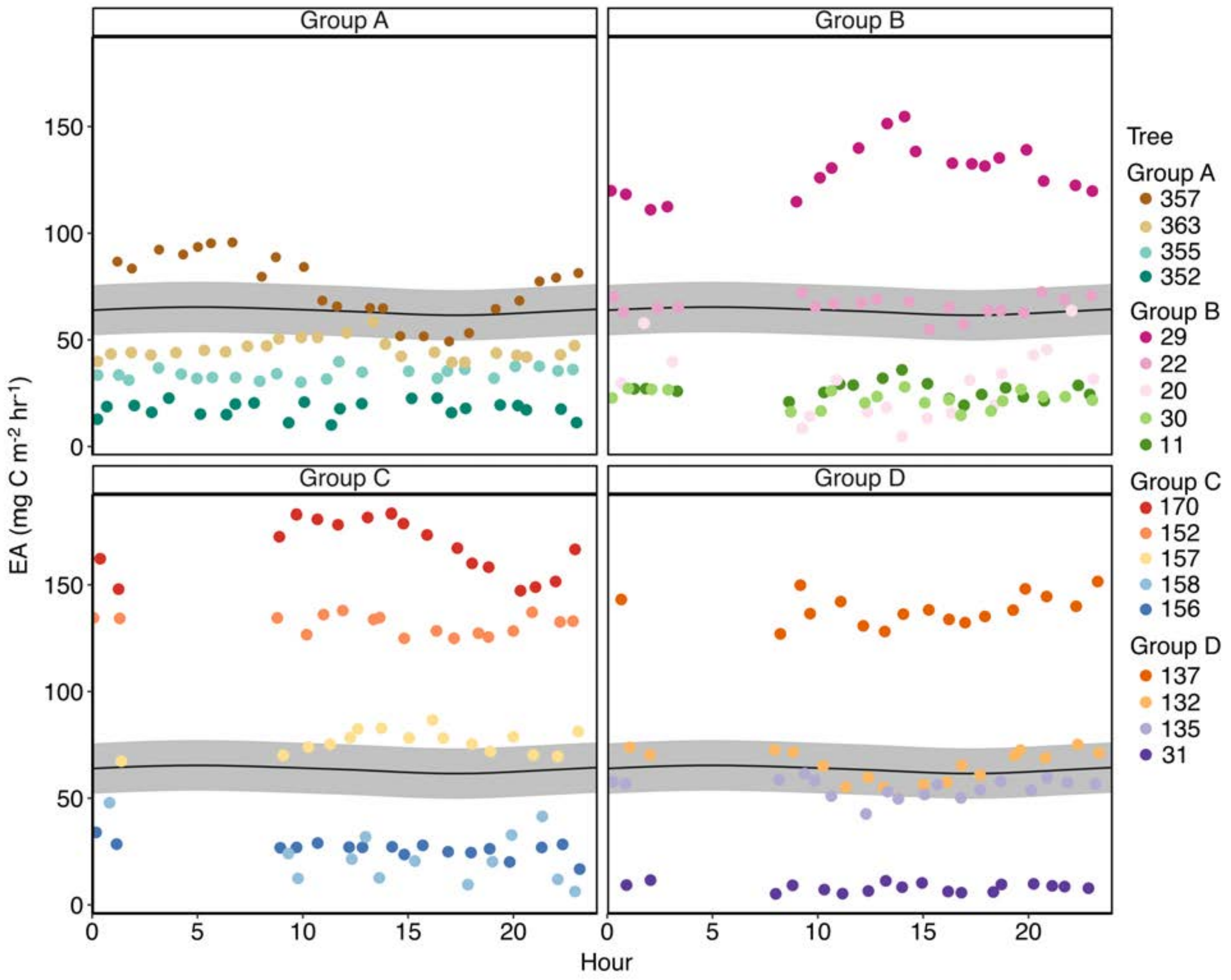

Figure S10: Diel normalized EA for the study trees. Each facet and color represent a different tree which can be identified by its tag number (colours also as per Figure S9). Solid lines show predicted EA for each tree from a generalised additive model (GAM) and dashed lines as the standard error of the prediction. The GAM model had normalized EA as the dependent variable, and hour by tree tag, fitted as a smoother with a cyclic cubic regression spline, and date-time (cyclic cubic regression spline) nested within tree tag (random effect) as a tensor smoother (to account for the autocorrelation within the dataset) as independent variables. Trees without a prediction line had no significant diel cycle. For model output see Table S2.

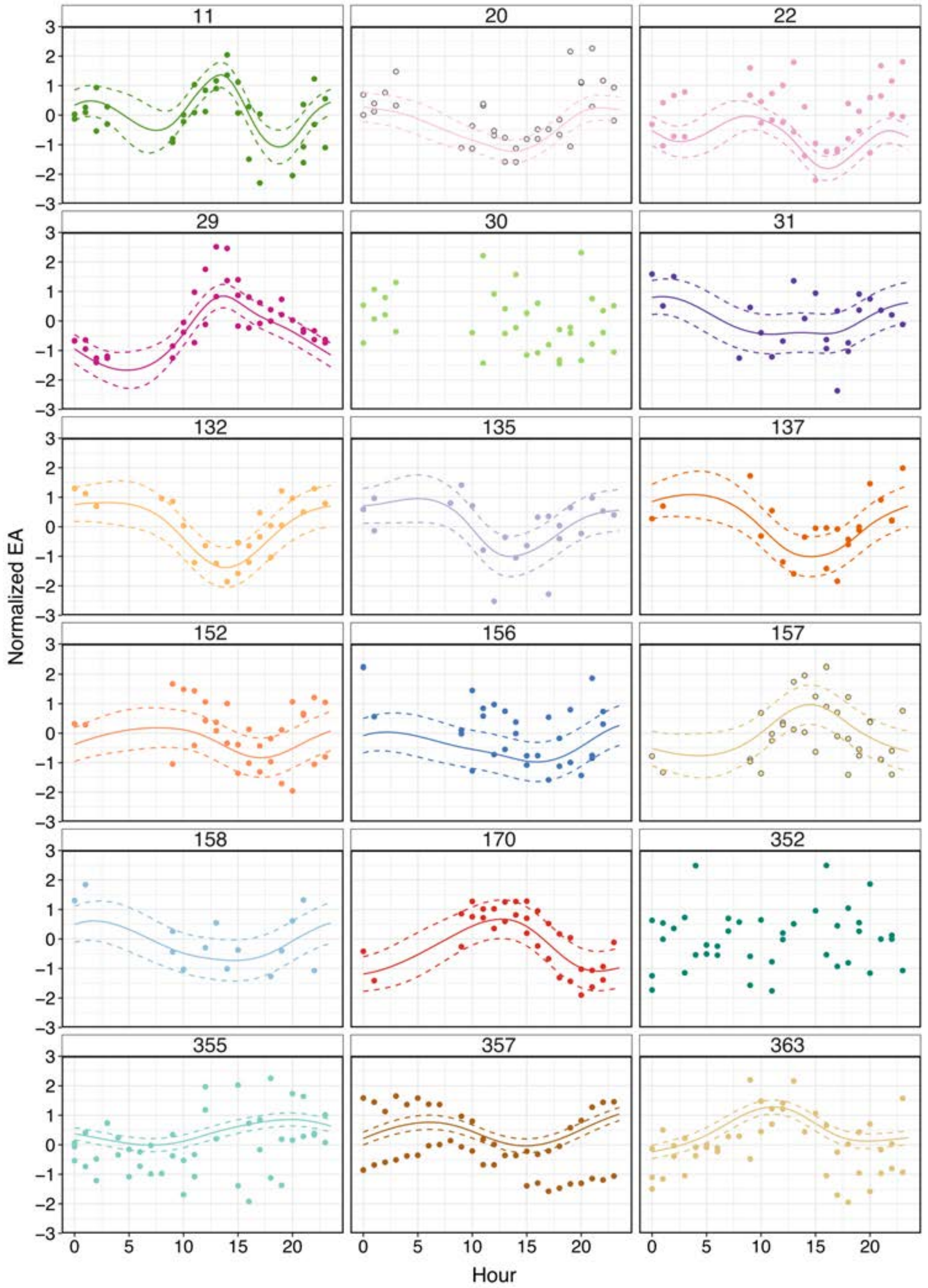

New Phytologist

Fig. S11: Boxplot of woody stem CO_2 efflux (EA) per unit stem area observations for measurements conducted on buttresses (green), stems (pink) and above the first major branching point (orange) during the vertical campaign. During this campaign, 15 trees were sampled, and EA was measured at minimum of four height intervals (range 4 – 7) per tree that were ~5 m or ~ 10 m apart, dependent on tree height. Overall, there was 68 observations of EA, with 6 measurements on buttresses, 49 on the stem, and 13 on the stem above the first branching point.

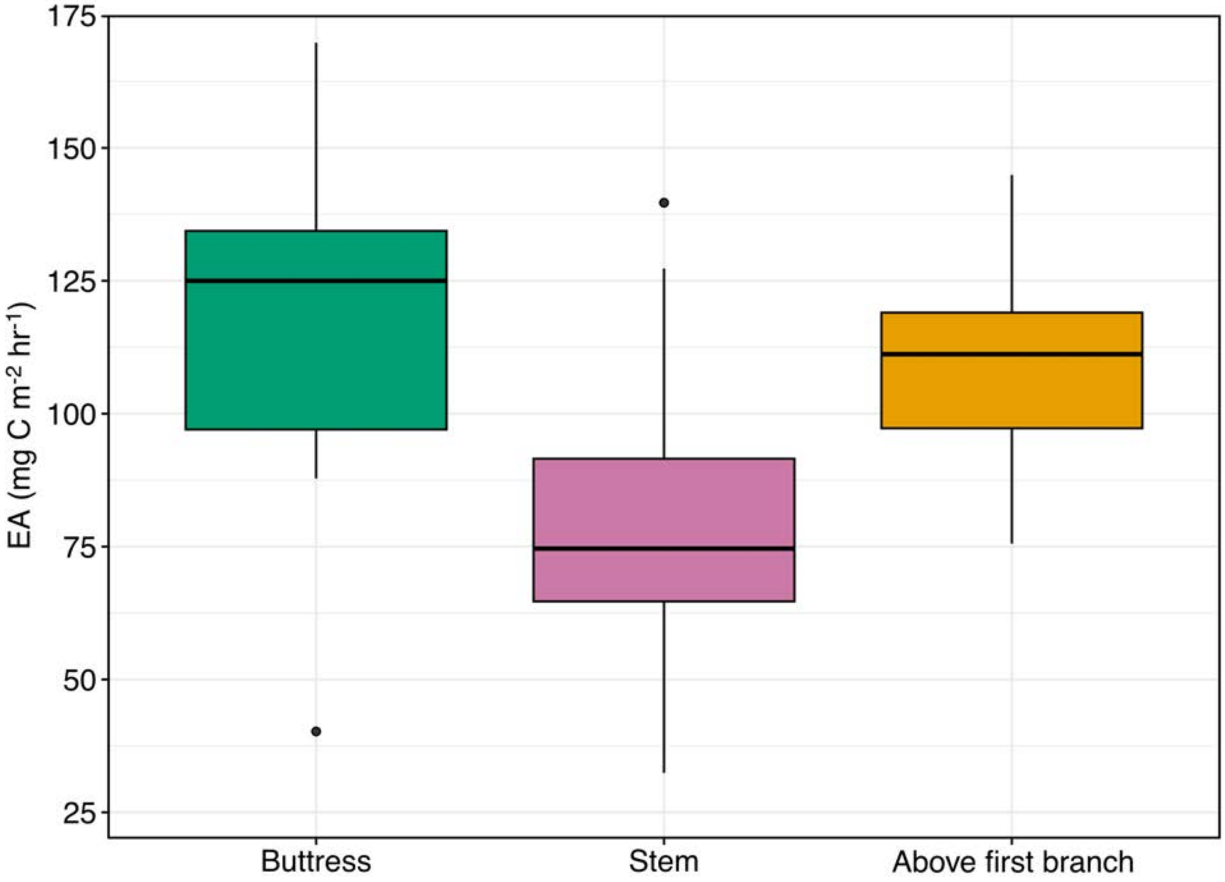

Fig S12: Woody stem CO_2 efflux (EA) per unit stem area along with tree proportional height (%). Points denote observed values (observations = 68, number of trees = 13), whereby the average observed EA has been calculated in 10 % proportional height intervals. Horizontal error bar represents standard error of the mean EA per height interval, vertical error bar shows the range of heights for each interval with point placed at the average height per interval. Black dashed line represents modelled vertical EA from a linear mixed effects model which was applied to all trees with terrestrial LiDAR scanned data with no branch truncation (n = 190). Average EA from modelled values was calculated at 0.25 % proportional height intervals and standard error for each interval (represented by bands). For both modelled and observed, green represents measurements on buttresses (triangles), pink represents measurements on the main stem (squares) and orange represents measurements above the first major branching point (circles).

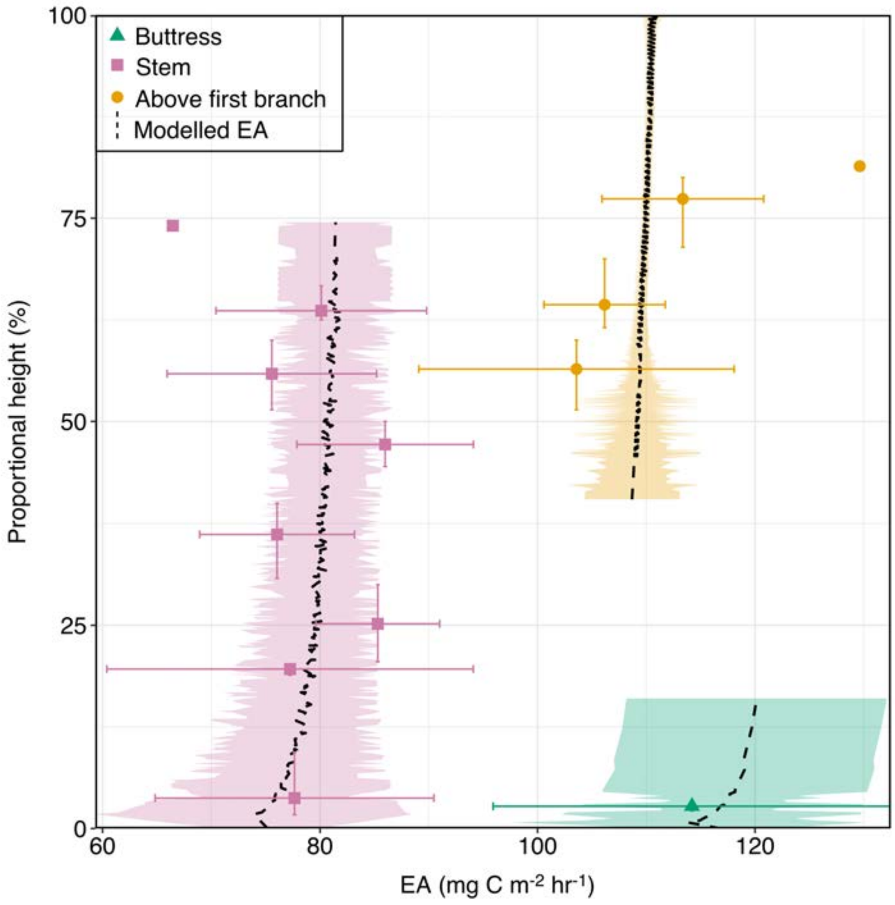

New Phytologist

Table S1: Models and Akaike information criterion created for vertical stem CO_2 efflux (EA) model, conducted in R package "nlme" (Pinheiro et al., 2023) using a mixed effects linear model. Whereby MH is height of EA measurement (m), P is position of measurement along the length of the tree (factor; stem, buttress or above the first major branching point), D is stem diameter (cm), and Tag is the tree tag number (factor).

Model	Akaike information criterion
**EA ~ log(MH) + P, random = ~ log(MH) | Tag**	**598.1322**
EA ~ log(MH) + log(D) + P, random = ~ log(MH) | Tag	599.6909
EA ~ log(MH) + log(D) + P, random = ~ log(D) | Tag	605.9969
EA ~ log(D) + P, random = ~ log(D) | Tag	605.3316
EA ~ log(MH) + log(D) + P, random = ~ Tag	615.3616
EA ~ log(MH) * log(D) + P, random = ~ log(MH) | Tag	600.4529

Table S2: Diel normalized EA model generalized additive model (GAM) output, whereby normalized EA was the dependent variable, and independent variables included hour by tree tag (fixed effect) fitted as a smoother with a cyclic cubic regression spline, and date-time (cyclic cubic regression spline) nested within tree tag (random effect) as a tensor smoother. The tensor smoother was included to account for the temporal dependence in the dataset, as trees were measured in groups on different days. Output shows the parameters, whereby s(Hour) indicated the smooth diel pattern for each tree, effective degrees of freedom (edf), reference degrees of freedom (Ref.df), F value and p-value.

Parameter	edf	Ref.df	F	p-value
s(Hour): Tree11	5.08	14	1.887	<0.001
s(Hour): Tree20	2.85	14	1.467	<0.001
s(Hour): Tree22	4.19	14	1.449	<0.001
s(Hour): Tree29	3.84	14	3.195	<0.001
s(Hour): Tree30	1.34	14	0.185	0.122
s(Hour): Tree31	2.42	14	0.687	0.005
s(Hour): Tree132	3.08	14	1.901	<0.001
s(Hour): Tree135	2.99	14	1.045	<0.001
s(Hour): Tree137	2.61	13	1.32	<0.001
s(Hour): Tree152	2.30	13	0.762	0.004
s(Hour): Tree156	2.19	13	0.574	0.015
s(Hour): Tree157	2.80	13	1.419	<0.001
s(Hour): Tree158	1.85	13	0.516	0.014
s(Hour): Tree170	2.75	13	2.633	<0.001
s(Hour): Tree352	0.00	14	0	0.406
s(Hour): Tree355	1.95	14	0.526	0.009
s(Hour): Tree357	2.52	14	1.106	<0.001
s(Hour): Tree363	2.93	14	1.365	<0.001
Tensor smoother:<br>Tree (random),<br>Date-time (cyclic cubic regression spline)	29.97	71	2.445	<0.001
Adjusted R^2 = 0.458,<br>Deviance explained = 53.1 %<br>REML = 728.24,<br>Scale est. = 0.53, n = 579				

Table S3: Wilcoxon signed-rank statistic scores to show if predicted values from diel GAM model (Table S2) were significantly difference from 0, which would indicate normalized EA is not equal to 0 and therefore measurements taken during these hours would not be representative of the median daily flux.

Hour	W	p-value
00:00	337	0.96
01:00	349	0.81
02:00	351	0.79
03:00	374	0.53
04:00	374	0.53
05:00	342	0.90
06:00	319	0.83
07:00	299	0.60
08:00	290	0.50
09:00	263	0.28
10:00	239	0.14
11:00	251	0.20
12:00	247	0.18
13:00	249	0.19
14:00	217	0.07
15:00	172	0.01
16:00	152	0.004
17:00	123	<0.001
18:00	127	<0.001
19:00	184	0.03
20:00	257	0.24
21:00	331	0.98
22:00	377	0.50
23:00	383	0.44

Table S4: Final model for vertical stem CO_2 efflux (EA) model with coefficients (and standard error in brackets), conducted in R package "nlme" (Pinheiro et al., 2023) using a mixed effects linear model. Whereby MH is height of EA measurement (m), P is position of measurement (factor; stem, buttress or above the first major branching point) and Tag is the tree tag number (factor). *** indicates $p<0.001$.

	Dependent variable:
	EA
log(MH)	1.669
	(3.604)
$P_{buttress}$	40.584***
	(11.711)
$P_{AboveBranch}$	28.695***
	(4.474)
Constant	75.662***
	(12.040)
Observations	68
Log Likelihood	-291.066
Akaike Inf. Crit	598.132
Bayesian Inf. Crit	615.888

Table S5: Total stem surface area (m^2) estimated for 190 trees by each estimation method. Whereby surface area has been estimated using an allometric equation from Chambers et al. (2004) (SA_a) and using an allometric equation with a site-specific correction coefficient of 1.669 (SA_{ha}) and from terrestrial LiDAR scanning with no truncation (TLS_{t0}) and with 2 cm truncation (TLS_{t2}) which is the inclusion and exclusion of branches with a diameter less than 2 cm, respectively. SA_a and SA_{ha} have a 10 % error assigned (Robertson et al., 2010) and TLS_{t0} and TLS_{t2} error was calculated by using the standard deviation for each tree (based on the surface area estimate of multiple tree QSM models per tree) using a Monte Carlo simulation.

**Surface area estimation method**	**Total surface area (m^2)**
SA_a	9453.58 ± 945.34
SA_{ha}	15776.87 ± 1577.70
TLS_{t2}	11305.93 ± 257.90
TLS_{t0}	15616.85 ± 236.63

Table S6: Stand-level woody stem CO_2 efflux (EA) as estimated by each surface area method and with vertical variation. In line with traditional methodologies, average EA at 1.1 m (95.77 ± 12.07 mg C m^{-2} hr^{-1}) was upscaled to the surface area of the plot. Surface area was estimated in four difference ways, whereby surface was estimated using an allometric equation from Chambers et al. (2004) (SA_a) and using an allometric equation with a site-specific correction coefficient of 1.669 (SA_{ha}), and using terrestrial LiDAR data with no truncation (TLS_{t0}) and with 2 cm truncation (TLS_{t2}) which is the inclusion and exclusion of branches with a diameter less than 2 cm, respectively. For this, surface area was calculated for 190 trees per each method and average EA at 1.1. m and standard error upscaled to the surface area. For TLS_{t2} and $TLS_{t0,}$ stand-level EA was also estimated including vertical variation, whereby the vertical model (Table S1) was applied to the TLS data and error propagated from the standard error predicted from the vertical model.

**Surface area method**	**Upscaled flux**	**Stand-level EA (kg C day^{-1})**
SA_a	1.1 m	21.75 ± 2.74
SA_{ha}	1.1 m	36.30 ± 4.57
TLS_{t2}	1.1 m	25.99 ± 3.28
	Vertical variation	27.57 ± 0.63
TLS_{t0}	1.1 m	35.90 ± 4.52
	Vertical variation	38.85 ± 0.59

Table S7: Model output for the linear mixed effects model used to determine the difference between total upscaled EA per tree from each scaling method and the interaction of including vertical variation. Within the model, upscaled EA per tree was the dependent variable and surface area method (SA_a, SA_{ha}, $TLS_{t0,}$ TLS_{t2}) interaction with vertical scaling (TLS_{t0} - V, TLS_{t2} - V) was the independent variables and tree tag as a random effect. Output shows the compared groups, t-ratio and p-value.

Compared groups		t-ratio	p-value
SA_a	SA_{ha}	-9.183	<0.001
SA_a	TLS_{t0}	-8.928	<0.001
SA_a	TLS_{t2}	-2.672	0.08
SA_{ha}	TLS_{t0}	0.255	0.99
SA_{ha}	TLS_{t2}	6.511	<0.001
TLS_{t0}	TLS_{t2}	6.256	<0.001
SA_a	TLS_{t0} - V	-10.789	<0.001
SA_a	TLS_{t2} - V	-3.669	0.004
SA_{ha}	TLS_{t0}- V	-1.606	0.59
SA_{ha}	TLS_{t2} - V	5.514	<0.001
TLS_{t0}	TLS_{t2} - V	5.259	<0.001
TLS_{t2}	TLS_{t0} - V	-8.117	<0.001
TLS_{t2}	TLS_{t2} - V	-0.996	0.92
TLS_{t0}	TLS_{t0} - V	-1.861	0.43
TLS_{t0} - V	TLS_{t2} - V	7.121	<0.001

References

Chambers JQ, Dos Santos J, Ribeiro G, Higuchi N. 2009. LBA-ECO CD-08 Tree Inventory Data, Ducke Reserve, Manaus, Brazil: 1999.

Chambers JQ, Tribuzy ES, Toledo LC, Crispim BF, Higuchi N, Santos JD, Arau´jo AC, Arau´jo A, Kruijt B, Nobre AD, *et al.* 2004. Respiration from a tropical forest ecosystem: partitioning of sources and low carbon use efficiency. *Ecological Applications* 14: 72–88.

Feldpausch TR, Banin L, Phillips OL, Baker TR, Lewis SL, Quesada CA, Affum-Baffoe K, Arets EJMM, Berry NJ, Bird M, *et al.* 2011. Height-diameter allometry of tropical forest trees. *Biogeosciences* 8: 1081–1106.

Pinheiro J, Bates D, R Core Team. 2023. nlme: Linear and Nonlinear Mixed Effects Models.

Wood SN. 2011. Fast Stable Restricted Maximum Likelihood and Marginal Likelihood Estimation of Semiparametric Generalized Linear Models. *Journal of the Royal Statistical Society Series B: Statistical Methodology* 73: 3–36.
